# Communication and health literacy in pediatric emergency: nursing team’s perspective*

**DOI:** 10.1590/1980-220X-REEUSP-2024-0294en

**Published:** 2025-06-06

**Authors:** Adélia Karla Falcão Soares, Francisca Márcia Pereira Linhares, Mirelly da Silva Barros, Fernanda Machado Silva-Rodrigues, Erika Acioli Gomes Pimenta, Queliane Gomes da Silva Carvalho, Maria Wanderleya Lavor Coriolano-Marinus

**Affiliations:** 1 Universidade Federal de Pernambuco, Programa de Pós-Graduação em Enfermagem, Recife, PE, Brazil.; 2 Universidade Federal de Pernambuco, Programa de Pós-Graduação em Saúde da Criança e Adolescente, Recife, PE, Brazil.; 3 Universidade de São Paulo, Escola de Enfermagem, São Paulo, SP, Brazil.; 4 Universidade Federal da Paraíba, Departamento de Enfermagem, João Pessoa, PB, Brazil.; 5 Universidade Federal de Pernambuco, Departamento de Enfermagem, Recife, PE, Brazil.

**Keywords:** Health Education, Health Communication, Health Literacy, Child, Hospitalized, Emergencies

## Abstract

**Objective:**

To analyze the nursing team’s knowledge and perspectives on an educational process focusing on communication and health literacy in the context of a pediatric emergency.

**Method:**

A qualitative study, based on participatory health research and the health literacy theoretical framework. The educational process was conducted in a pediatric emergency room between October and November 2021, with the participation of ten nurses and 28 nursing technicians. Data were collected from operational groups’ interactions and productions during the educational process and analyzed through descriptive and analytical coding, with the support of Atlas.ti software version 8.0.

**Results:**

Participants recognized barriers in communication with children and their families, expanding their repertoire of communication strategies, reinforcing the use of therapeutic toys, the teach-back technique and puppets.

**Conclusion:**

Teaching-learning activities encouraged the nursing team’s active and reflective participation, expanding knowledge and health literacy skills.

## INTRODUCTION

Health literacy (HL) is defined by the World Health Organization as the “ability to obtain, process, and understand health information to make decisions about self-care”^(1)^. It is a social determinant of health influenced by structural, situational and contextual conditions that can limit, favor or modify children’s and adolescents’ capacity to act in healthcare contexts. This process is shaped by the social environment in which they are inserted, including relationships with caregivers, friends, teachers, healthcare professionals, among others^(2)^.

The pediatric emergency room is a critical care environment that can be perceived as impersonal, with restrictions on children’s daily activities (such as interacting and exploring the various spaces around them). This makes the care provided by healthcare professionals a challenge, as it can represent a momentary or lasting threat to pediatric patients’ physical and emotional integrity, especially when their needs are not understood and specific strategies for comprehensive care are not used^(3)^.

Emergency room staff deal with trauma, illness and fatalities that often make the setting chaotic, and establishing good communication can be difficult^(4)^. Therefore, in addition to technical and emotional care, professionals need to establish bonds of trust with patient and family as well as improve the communication and HL process with children, considering their particularities.

The nursing team constitutes the main group of professionals in continuous care, carrying out different strategies to assist hospitalized children and their caregivers, through health education^(3,5)^. Some factors may limit the understanding of information related to children’s health by parents or caregivers, such as level of education and schooling, low HL, anxiety, concern about children’s suffering, and inadequate or inefficient communication during hospitalization^(6)^. Nursing professionals’ barriers and limited communication skills can interfere with their ability to interpret pediatric patients’ verbal and nonverbal communication, in addition to making it difficult for caregivers to assimilate information shared by their health status^(4)^.

Communication between nursing staff and caregivers should promote a relationship of empathy, support and trust, which is essential for establishing a bond between the subjects involved. Moreover, it is a tool to reduce fear and anxiety in pediatric patients and caregivers, making emergency care less traumatic^(7)^. From this perspective, it contributes as a care tool for HL, which emerges as an intermediary construct of educational activities in healthcare services^(1)^. The 6D (six dimensions) model of HL for children and adolescents involves particularities related to child development and social determinants of health. The appropriation of knowledge and tools focused on HL can increase children’s leading role in the sense of accessing, understanding, assessing and applying information based on interaction with healthcare professionals^(8,9)^.

Studies point to the relevance and effectiveness of interventions with elements that aim to increase empathy and communication between healthcare professionals and children. To achieve these objectives, there is a need for training in communication strategies that qualify the nursing team to promote HL for children and their caregivers^(4,7)^. The use of tools focused on HL from an early age is considered a promising investment in the health and well-being of these patients, both now and throughout their adolescence and adult life^(8)^.

Given the concepts of health communication as an activity to improve HL presented here, it becomes crucial to invest in training actions to ensure that healthcare institutions are responsive to HL^(10)^. In view of this, continuing education in health is a space for dialogue and problematization of everyday life, as long as professionals are encouraged to rethink their performance in a permanent process of qualification. This movement should promote changes that improve the quality and effectiveness of healthcare for the population^(5)^.

Based on the potential of continuing education for changes in professional practice and the process of reflection on practice, this study aimed to analyze the nursing team’s knowledge and perspectives on an educational process focused on communication and HL in the context of a pediatric emergency.

## METHOD

### Study Design

This is a qualitative study with a methodological framework in participatory health research, a modality based on the interrelationship between the actors and knowledge involved in a social and dialogical practice from Paulo Freire’s perspective^(11)^, which allows the inclusion of participants’ experiences and reflections^(12)^. This participatory research was carried out based on an educational process on communication and HL with the nursing team of a pediatric emergency department. This article followed the guidelines proposed by the COnsolidated criteria for REporting Qualitative research (COREQ) guide^(13)^.

The research was carried out during the context of the COVID-19 pandemic, respecting current protocols, such as the distancing of participants in the room and the use of masks.

### Population and Sample

Sample size was intentionally defined based on all eligible participants, invited through in-person visits conducted by the researcher and via WhatsApp^®^ and Instagram^®^. All interested parties were informed by the researcher that this was a research based on an educational process carried out in the pediatric emergency room, including synchronous and asynchronous activities, with a total workload of 40 hours and delivery of a certificate of participation upon completion. In the initial recruitment, 44 nursing professionals agreed to participate, ten nurses and 34 nursing technicians. However, six nursing technicians did not attend any meeting due to personal reasons and unavailability to participate in the proposed activities. Thus, the final sample consisted of 38 professionals, ten nurses and 28 nursing technicians.

### Selection Criteria

Inclusion criteria considered pediatric emergency nursing professionals on day shifts, who developed direct care actions for children aged 0 to 13 years, 11 months and 29 days, with at least one year of experience in the role and availability to participate in the educational process and all activities proposed during the work shift. Exclusion criteria considered professionals who were on leave during the period of the educational action.

### Study Setting

The educational process was carried out in a Brazilian public hospital in the city of Recife, PE, a reference in pediatric emergency care for children aged 0 to 13 years, 11 months and 29 days, with medium and high complexity conditions. The hospital receives cases referred and regulated by the public health network of the state of Pernambuco.

### Educational Process Description

Educational process planning was prepared with the intention of incorporating precepts of action-reflection-action present in the Brazilian National Policy for Continuing Education in Health^(3)^. The teaching-learning strategies involved active methodologies, with an emphasis on the theoretical framework of experiential learning developed by David Kolb^(14)^.

To ensure greater feasibility of professionals’ participation, the educational process was carried out during the daytime work shift, from 10:00 to 11:30 (morning) and 13:00 to 14:30 (afternoon). To meet the needs of all participants, meetings were repeated three times, before and during work breaks, adjusting to the service dynamics.

After registering and signing the Informed Consent Form (ICF), participants participated in all activities of the educational process. In the first meeting, two questions were presented: 1) What are your expectations for this training on HL and communication with children? 2) What reasons led you to choose to participate in this educational process?

The educational process was structured in four meetings, each with a specific topic: Topic 1: Recovering nursing professionals’ inner child; Topic 2: Types of communication approaches with children/families in a hospital environment; Topic 3: Communication and health literacy in professional practice; Topic 4: Perceptions about the communicative approaches implemented to promote health literacy in the workplace (Chart 1).

**Chart 1:** Topics and educational strategies used in group sessions – Recife, PE, Brazil, 2021.

Group sessions	Educational process
TOPIC 1 – Recovering nursing professionals’ inner child	**Awareness-raising dynamics on the topic:** drawings that marked childhood between the ages of 6 and 12. Who is this inner child? How has this inner child communicated with the children under my care in the emergency room? **Dialogical exposure**: knowing the physical, emotional and social aspects of the child. Images and assumptions from the book “The Whole-Brain Child” (Daniel Siegel and Tina Payne).
TOPIC 2 - Types of communication approaches with children/families in a hospital environment	**Dialogical exposition:** based on a literature review on the types of communication approaches with children/families in the context of hospitalization. Illustrate communication strategies with approaches already used in the service. **Guest:** Mirelly Barros (Master’s student in the Graduate Program in Child and Adolescent Health at the *Universidade Federal de Pernambuco*). Participation via the Google Meet^®^ platform with the topic “Storytelling as a communication strategy with children in the context of hospitalization”. Narration of the story “The Heart and the Bottle” (Oliver Jeffers).
TOPIC 3 - Communication and health literacy in professional practice	**Present the concepts of communication and HL. ROLE PLAYING - Simulation built from David Kolb’s Experiential Learning Theory. Practical application (pair or trio):** participants created two situations involving the nursing team, with the purpose of articulating the dimensions of HL and communication approaches. In these activities, empathic communication and HL promotion strategies were used.
TOPIC 4 - Perceptions about the communicative approaches implemented to promote health literacy in the workplace	**Recovering teaching-learning activities in the workplace:** practical application of one of the communication strategies and articulation with the HL concept (three to five-minute filming and self-reflection).
Participants’ assessment and self-assessment.	**“Tree of Knowledge” dynamic:** to explore participants’ impressions about the application of communicative approaches in promoting HL in pediatric emergency care.

Source: prepared by the author.

Each meeting had the following pedagogical sequence: 1) experiential dynamics to identify previous knowledge and experiences on the topic; 2) approach of the main topic by a moderator, introducing new knowledge; 3) self-reflection questions shared in the group; 4) suggestions of practical activities for the work environment, encouraging participants to bring feedback to the subsequent meeting.

At the end of each meeting, a question was asked to assess information about participants’ understanding and perception of each topic.

### Data Collection

To collect data, the operative group (OG) technique was used, which places participants at the center of the learning process, making them leading actors in the construction of knowledge^(15,16)^. The group sessions took place between October and November 2021, organized as follows: session opening; introduction of participants to each other; clarification of participatory discussion dynamics; setting establishment; debate; synthesis; and session closing.

The data collection instruments consisted of recordings of group interactions. Such communications, mediated by the guiding questions recorded in the educational process description section, were the main *corpus* of analysis for this work.

In total, 12 OG sessions were held, each lasting approximately one and a half hours and attended by four to ten participants, considering an average of eight to ten professionals^(15)^. The OGs were led by a graduate student moderator, with theoretical and practical training in qualitative research, and two to three undergraduate students, who received prior training to conduct and support the activities.

The moderator was responsible for the preparation, organization and instrumentation at all stages, while observers accompanied the sessions and recorded participants’ statements in field notes, in addition to assisting in audio handling and image recording equipment. The total number of assistant members was nine observers.

The chosen location for the OGs was the hospital’s own classroom, which offered adequate structure, lighting, and temperature, providing a private and welcoming environment. The seats were arranged in a semicircle, ensuring that the nursing staff, moderators, and observers shared the same field of vision. Due to the pandemic, sanitary measures were adopted to ensure the safety and well-being of researchers and the target audience.

### Data Analysis and Treatment

The narratives were recorded in audio and video, with the consent of participants. The discursive pauses and intonations captured by the moderator and observers, as well as facial expressions, gestures and other body language, were recorded in “field notes”. At the end of the educational process, the data were transcribed in full and submitted for reading and validation by participants, who could make changes if necessary.

The *corpus* analyzed was based on the narratives produced throughout the educational process and on field notes, examined through qualitative coding, following the stages proposed by Graham Gibbs (2009): line-by-line coding; categorization of topics; and data analysis^(17)^. To assist in thematic coding and categorization process, Atlas.ti software (version 8.0) was used, which allowed the segmentation of the collected material into descriptive codes, code groups and final categories. Coding was carried out by the first author (AKFS) together with the advisor (MWLCM), and data synthesis was articulated with the HL framework.

### Ethical Aspects

The study followed all legal guidelines and prerogatives established by Resolutions 466/2012 and 510/2016 of the Brazilian National Health Council, and was approved by the *Universidade Federal de Pernambuco* Research Ethics Committee, according to Opinion 4,755,034. All participants received guidance and clarifications about the study and were then given the ICF, duly signed after accepting to participate in the research. Participant confidentiality and anonymity were guaranteed, identified by means of an alphanumeric system composed of the letters “N” (Nurses) and “T” (Nursing Technicians) and the number corresponding to the order in which participants completed the ICF.

## RESULTS

The results were obtained from 38 professionals, ten nurses and 28 nursing technicians, the majority of whom were female (95.46%), aged between 26 and 62 years. These professionals had, on average, 17 years of training and five years of experience in the institution. Concerning qualifications, three nurses (30%) and three nursing technicians (10.7%) had specializations in their field of activity, and four nurses (40%) had master’s degrees. At the end of the educational process, six nurses and 18 nursing technicians completed 70% of the proposed activities, totaling 24 participants.

From the *corpus* coding, two thematic categories were systematized: 1 – Nursing team’s perceptions and communication strategies; 2 – Knowledge and perspectives of the educational process based on communication and health literacy skills.

### Category 1- Nursing Team’s Perceptions And Communication Strategies

#### Barriers to Communication with Children

In their statements, participants mentioned barriers to communicating with children, including the need to understand physical and emotional aspects and children’s level of development. The pediatric emergency environment, limited time for care, and caregivers’ literacy also made communication difficult. Furthermore, it was clear that the severity of children’s condition influenced the communicative approach in an emergency setting.

*We know that it is a pediatric emergency (...) especially in the stabilization room, you do not have more time to calm down, to organize, to exchange that empathetic look with the child (...) unlike the green ward and the yellow ward where you can, which is not an emergency child, who is not in an emergency situation, and there you can have more time with that child, despite the demand. I visualized these barriers: service, the service setting, the child depending on the stage of the disease in which they are and the parents’ level of education.* (N02)

Participants identified feelings such as fear of the unknown in children and expressed disagreement with the frequent practice of “holding children down by force” during procedures. This behavior was seen as an obstacle to the relationship of trust and communication between the nursing team and the dyad.

*At first, the child is very afraid! Just hearing (the word - author’s addition) hospital scares them, and it depends a lot on the mother or the person accompanying them. It is important to approach this child and talk to them; it depends on the child’s age and the context, but fear is really a barrier (...).* (T17)

*I’ve been here for 4 years, and I don’t accept that some buco (oral and maxillofacial surgeons - the term was added by the author), orthopedists, and doctors have to suture the child by force, you know? I think this causes unnecessary trauma to the child, so the message that is left to that child is that the healthcare professional is a bad, evil person; they are perverse, they are the one who hold the child by force and hurt.* (N07)

#### Communicative Strategies in the Relationship with Children

Strategies highlighted by participants in their experiences with children included the use of playful language, cartoons and personalized uniforms with childlike motifs, aiming at distraction and interaction during procedures such as dressing changes, venipuncture and rectal lavage.

*It was a child who was going to have a rectal lavage, and another technician was going to do it. But the Frozen T12 shirt caught so much attention that the child said, “I want Frozen!”. So, like it or not, this brings the child closer and breaks the fear.* (N06)

*So, I started trying to talk to him. He was wearing a Spider-Man shirt, so we took that and tried to bring up a subject that might be of interest to him, because sometimes it’s a song, a character that’s on the shirt (...) so it’s a way for us to break the ice and start communicating with that child (...).* (N07)

*I said, “It’s a very bearable little prick,” like an ant that we feel and don’t cry. That’s it, this prick is the same thing, then he said, “Can you show me the size of this needle?” (...) then I showed him the size of the Jelco, the little yellow one, 24. See what happened in his mind, “Hey, auntie, what are the size of the babies here? Because he said the needle was big”.* (T07)

Therapeutic play was cited as a communication strategy with children. For participants, effective communication involves welcoming and developing trust, allowing access to health information during care actions.

*But I think there is something called “therapeutic play” where we always try to explain to the child what is going to be done to them, and it works for any procedure; it relieves a lot of tension. However, you need some material for this: a doll, material to simulate the tube and the syringe (...) to get close, touch and have a moment with the child (...).* (N14)

#### Communication Strategies with the Family

Regarding communication strategies with the family, participants emphasized the importance of a more empathetic welcome, such as the use of non-verbal communication, in which facial expressions and body language act as facilitators or hindrances to this welcome during children’s hospitalization.

*I think we have to be very patient, know how to speak (...). You can say no in many ways, you can say no rudely or you can say no delicately (...), the way you say it, the tone you use. Because many times the body speaks, the body certainly speaks! Your facial expressions and the movements of your body, so (...).* (N08)

Participants perceived that socioeconomic issues and the educational level of caregivers/families accompanying children in the emergency room interfered with the understanding, assessment and application of health information. Moreover, they recognized the lack of tools to use simpler and more accessible communication.

*The first point, I believe, is the parents’ level of education, and I realize that the lower the level of education, the more insecurity it generates in this process of learning about the child’s illness and it is more difficult to talk to this person, because we are not prepared after we graduate to stop using technical terms (...). It is not regressing, but speaking in a way that the population understands.* (N02)

*I am with that patient’s mother, and I see that she has no quality of understanding, and I give myself until they can understand what I am saying. I give myself with compassion. I put myself there in that place.* (T07)

### Category 2 - Knowledge and Perspectives of the Educational Process Based on Communication and Health Literacy Skills

#### Access to Health Information

Participants highlighted that, in addition to understanding children’s physical and emotional aspects, it is important to involve the child in the care process, ensuring that they have access to clear and truthful information about procedures and treatments. Furthermore, they emphasized that professionals should do the same in relation to caregivers, asking for their support.

*Because, if we are going to puncture, we know that they will cry, scream, and run, but first of all, we have to talk and explain what we are doing, even when they often don’t want to pay attention, but it is always good to explain. Asking the mother to help is also very important (...). The mother calms the child while we do our work.* (T06)

#### Understanding and Assessing Health Information

Participants applied communication strategies learned in the educational process, such as the use of puppets. This experience allowed them to identify the feelings of children and their mothers, in addition to highlighting the potential of play to reduce pain and fear. They also recognized the importance of peer support in teamwork.

*In fact, we wanted to take a big child because we thought she wouldn’t cry (...) but the girl screamed, screamed a lot, because she had a lot of abdominal pain (...) we tried to calm her down somehow, and brought the puppets to distract her (...). The mother was very anxious, because she didn’t want the child to go into surgery, and we somehow tried to calm the mother down, medicated the girl, anyway, when we passed the shift, the girl was sleeping very peacefully (...).* (T14)

*(...) I can approach her (the child - author’s addition) and explain, from the beginning, how that approach will be, to reassure her, to bring the family in as a helper in this process, because every time we approach to administer medication, a procedure, that child understanding my communication with them will not be afraid (...).* (N01)

Another strategy used by participants was the teach-back technique, applied to explain and reinforce health information and treatments directed at children in pediatric emergencies to family members.

*When I was in the course, I went to learn more about the subject, and I saw that the teach-back technique is the final stage in terms of health communication. I saw that it is extremely important because it generates benefits in the medium and long term. This approach is the main link with the companion, because we really need their help*. (T02)

*Communication, I think, in the case of literacy, is the act of making oneself understood, because there is no point in me speaking if the person receiving the information does not understand what I am saying. So, there was that barrier, there was that filter, they were unable to understand.* (N08)

#### Application of Health Information

Participants shared their impressions and contributions to the educational process, highlighting the increased ability to understand the pain and needs of children and their caregivers. The practical application of communication strategies in daily life was also observed, taking into account the perspectives of children and their families.

*For me, the course helped me to improve my communication, patience and empathy with the mother and child, to show them the simplest things, to observe the context of their lives more closely, as this makes it clearer for them and for me, to help in the child’ daily care.* (T19)

*The way of approaching families helped me a lot, and now we know more clearly how to do this approach, the language, the way of speaking (...) as far as the person can understand in a clear and objective way.* (T06)

A highlight was the perception of the need for continuous processes of continuing education in professional practice as a resource to promote the expansion of knowledge, skills and reflections.

*(...) this type of course is not for you to participate once a year; this is continuing education, and when we come and forget, we have new training, we rescue (...) this issue of health education is important at all levels of care; this is patient safety, so this (literacy - author’s addition) cannot be a one-off education, it has to be continuous education (...).* (N08)

## DISCUSSION

Based on the objective of analyzing the nursing team’s knowledge and perspectives regarding an educational process focused on communication and HL in the context of a pediatric emergency, it was identified that nursing professionals recognized the barriers that prevented empathetic and sensitive communication with children and, consequently, the expansion of a more sensitive view regarding the needs of children and families treated in the pediatric emergency. The reflective activities, combined with practical activities, provided elements for health communication that generated greater satisfaction in the world of work and, additionally, encouraged the cooperation of children and family members as allies in nursing care.

Communication is the basis of HL, essential for establishing relationships of trust between people^(18)^. Despite the uncertainties and stress present in the practice circumstance, participants shared their knowledge and experiences, which contributed to the reflection and improvement of communication and HL skills in the hospitalization context.

The pediatric emergency scenario is often difficult and chaotic, and can represent a barrier/filter in the communication process, as studies point out^(19)^. In this regard, the “fear of the unknown” demonstrated by children has been identified as one of the main elements that hinder communication. In addition, other studies confirm that pediatric patients may fear healthcare professionals, associating their presence with painful procedures^(2,20)^.

The reports pointed to experiences in verbal and nonverbal communication that harm the quality of child care, such as the practice of holding children “by force” during procedures in the pediatric emergency room. According to participants, this behavior negatively impacts the way in which children cope with hospitalization, as they recognize the importance of respecting their feelings, desires and particularities. Studies highlight the importance of giving visibility to the active role of children in HL, ensuring their voice and understanding their needs through strategies that promote HL^(21,22)^.

The nursing team also identified other communication barriers, such as socioeconomic aspects, educational level and the physical and emotional state of caregivers/families during hospitalization. Research highlights that demographic, socioeconomic factors and the family’s HL level influence children’s health status and behavior, impacting their HL skills^(21,23)^.

The lack of communication and health information during child care by the nursing team can generate conflicts and distress in caregivers/family members. The way they deal with treatment influences child participation as well as their understanding of health information and the application of these concepts in their self-care^(8,24)^.

The creation of bonds between the nursing team and the family from the beginning of child hospitalization is essential to alleviate the stress caused by procedures and treatments. To achieve this, healthcare professionals must combine broad technical skills with knowledge and abilities in the areas of health communication, conflict mediation, problem solving, health marketing and creativity. In addition to this, it is essential to have social skills that promote a more humane, holistic and indepth approach to patients, considering their context^(9)^.

Regarding communication strategies with children, some participants reported the use of uniforms with children’s topics and the adoption of playful language to demonstrate the materials that would be used in dressing changes and venipuncture. Communication based on comparison and distraction, using cartoon images during procedures, allowed the nursing team to interact with children, respecting their level of cognitive and socio-emotional development^(25)^.

Our perception of children and young people is deeply linked to our subjective view of childhood and the social role attributed to this group in everyday interactions. In this context, promoting HL in children and families requires the improvement of HL skills with a focus on personal attributes, considering three central categories: cognitive (understanding and assessment); behavioral/operational (access and application); and affective/conative (motivation). These skills interact with social and environmental factors and are developed throughout the different stages of childhood and life^(8,26,27)^.

From the HL perspective, children’s emotional and social needs must be met by the nursing team through strategies that make them an active subject in their care process^(18,28)^. Some professionals highlighted the use of therapeutic toys - a facilitating approach that uses a structured toy to minimize pain and anxiety - as a resource to explain procedures to children, facilitating their adaptation to experiences in the hospital environment^(28)^.

A study on the nursing team’s perception of the benefits of playful strategies revealed that games, puppets, storytelling, accessories with children’s topics and music can favor children’s adaptation, participation and collaboration during hospital procedures^(25)^. Participants experienced and perceived the knowledge and perspectives of the educational process on communication and HL during teaching-learning activities in the workplace. Some used puppets as a playful strategy to guide children diagnosed with appendicitis and their family, explaining to them the preparation and performance of a surgical procedure.

Research carried out in Brazil in 2022 confirms that the use of playful strategies, such as improvisation and personalization of dolls and puppets, facilitates communication between nursing professionals and hospitalized children^(16)^. By adopting a language appropriate to childhood, professionals promote interaction and active participation of children, allowing them to express their concerns, fears and points of view. This approach helps to reduce emotional distress and minimize hierarchical relationships between children and adults^(22,24)^.

Another communication and HL strategy adopted by the nursing team was the teach-back technique, whose main objective is to explain health information to children’s caregivers and family members and to check their understanding of what was transmitted. This method, based on reteaching, can improve the understanding of those involved and reduce medication errors that lead to new readmissions^(4,29)^. Applying teach-back routinely is an effective alternative to HL-based screening, providing healthcare professionals with relevant, cumulative and up-to-date information on this subject and allowing a deeper understanding of caregivers/family HL needs^(29)^.

Participants’ reports showed that communication is an essential link in promoting HL skills (accessing, understanding, assessing, and applying health information) in hospitalized children and their caregivers. Research conducted in the United States in 2019 highlights that HL transcends individual skills, being strongly associated with the way information and care are offered by health providers^(4,30)^. In this sense, participants observed that the educational process on HL must be incorporated into the different levels of care, be continuous and made available to all healthcare professionals working in the service.

Therefore, adopting communicative approaches that involve children in their self-care - prioritizing the physical and socioemotional aspects of childhood - will allow nursing professionals to develop simple, empathetic, welcoming and humanized communication in pediatric emergency care actions. Therefore, the simpler and more appropriate the communication between healthcare professionals and caregivers/family, the easier it will be for them to understand and access health information during children’s recovery and treatment^(1,24)^.

Limitations of this study include participants’ immediate assessment and attendance at the end of the educational process, influenced by the shortage of personnel, the nature of the service and the high work demand. External factors interfering in critical hospitalization environments and the pandemic scenario with the new coronavirus may have impacted nursing professionals’ involvement and participation in the activities carried out during the educational process.

Professionals’ engagement in continuing education activities, which require more time for reflection and knowledge building, appears to be a challenge to ensure greater external validity of the data. Another limitation was the analysis of communication only from nursing professionals’ perspective, although the importance of assessments centered on the child and family is recognized to assess the impact of the actions of other healthcare professionals in this scenario.

## CONCLUSION

This research provided relevant information about nursing professionals’ knowledge and perspectives regarding an educational process focused on communication and HL in the context of a pediatric emergency. As observed, nursing professionals’ communication can influence the child/family’s ability to understand, assess and apply health information to promote HL.

In addition to the playful strategies already used in pediatric emergency care, such as cartoons, accessories and uniforms with children’s motifs, participants expanded their communication skills based on HL, such as reinforcing the use of therapeutic toys, the teach-back technique and puppets. These strategies engage with children and motivate them to participate in care actions.

The teaching-learning activities encouraged the nursing team’s active and reflective participation, with a broader repertoire of HL knowledge and skills, especially regarding specific communication strategies and approaches for children. Furthermore, they strengthened the relationship of trust between professionals and the child/family. The data presented can support the application of teaching-learning methodologies in other pediatric emergency services as well as in other care environments in which health communication should be problematized and expanded through reflection on the work.

## Data Availability

The data supporting the results of this study were published in the dissertation that gave rise to the article, available at: https://repositorio.ufpe.br/handle/123456789/46079.
