# Supplementary material for: Communication and health literacy in pediatric emergency: nursing team’s perspective
Source: Rev Esc Enferm USP. 2025 Jun 6;59:e20240294. doi: 10.1590/1980-220X-REEUSP-2024-0294en (PMC12172668; doi:10.1590/1980-220X-REEUSP-2024-0294en)
Supplement: Translation

# Translation: Comunicação e letramento em saúde na emergência pediátrica: perspectivas da equipe de enfermagem*

**DOI:** 10.1590/1980-220X-REEUSP-2024-0294pt

**Authors:** Adélia Karla Falcão Soares, Francisca Márcia Pereira Linhares, Mirelly da Silva Barros, Fernanda Machado Silva-Rodrigues, Erika Acioli Gomes Pimenta, Queliane Gomes da Silva Carvalho, Maria Wanderleya Lavor Coriolano-Marinus

**Affiliations:** 1 Universidade Federal de Pernambuco, Programa de Pós-Graduação em Enfermagem, Recife, PE, Brasil.; 2 Universidade Federal de Pernambuco, Programa de Pós-Graduação em Saúde da Criança e Adolescente, Recife, PE, Brasil.; 3 Universidade de São Paulo, Escola de Enfermagem, São Paulo, SP, Brasil.; 4 Universidade Federal da Paraíba, Departamento de Enfermagem, João Pessoa, PB, Brasil.; 5 Universidade Federal de Pernambuco, Departamento de Enfermagem, Recife, PE, Brasil.

**Keywords:** Educação em Saúde, Comunicação em Saúde, Letramento em Saúde, Criança Hospitalizada, Emergências

## Abstract

**Objetivo:**

Analisar os conhecimentos e perspectivas da equipe de enfermagem sobre um processo educativo com foco na comunicação e letramento em saúde no contexto de uma emergência pediátrica.

**Método:**

Estudo qualitativo, baseado na pesquisa participante em saúde e no referencial teórico do letramento em saúde. O processo educativo foi conduzido em uma emergência pediátrica entre outubro e novembro de 2021, com a participação de dez enfermeiros e 28 técnicos de enfermagem. Os dados foram coletados a partir das interações e produções dos grupos operativos durante o processo educativo e analisados por meio de codificação descritiva e analítica, com apoio do *software* Atlas.ti, versão 8.0.

**Resultados:**

Os participantes reconheceram as barreiras na comunicação com as crianças e suas famílias, ampliando seu repertório de estratégias comunicativas, reforçando o uso do brinquedo terapêutico, da técnica *teach-back* e de fantoches.

**Conclusão:**

As atividades de ensino-aprendizagem estimularam a participação ativa e reflexiva da equipe de enfermagem, ampliando conhecimentos e habilidades de letramento em saúde.

## INTRODUÇÃO

O letramento em saúde (LS) é definido pela Organização Mundial da Saúde como a “capacidade de obter, processar e compreender as informações em saúde, no intuito de tomar decisões apropriadas para a gestão do autocuidado”^(1)^. É um determinante social da saúde influenciado por condições estruturais, situacionais e contextuais que podem limitar, favorecer ou modificar a capacidade de ação de crianças e adolescentes em contextos de atenção à saúde. Tal processo é moldado pelo ambiente social em que eles estão inseridos, incluindo as relações com cuidadores, amigos, professores, profissionais de saúde, entre outros^(2)^.

A emergência pediátrica é um ambiente crítico de cuidados que pode ser percebido como impessoal, com restrições às atividades diárias da criança (como interagir e explorar os diversos espaços ao seu redor). Isso torna a assistência prestada pelos profissionais de saúde um desafio, pois pode representar uma ameaça momentânea ou duradoura à integridade física e emocional do paciente pediátrico, especialmente quando suas necessidades não são compreendidas e não se utilizam estratégias específicas para um cuidado integral^(3)^.

A equipe da sala de emergência lida com traumas, doenças e fatalidades que tornam o cenário comumente caótico, e estabelecer uma boa comunicação pode ser difícil^(4)^. Desse modo, além dos cuidados técnicos e emocionais, o profissional precisa estabelecer vínculos de confiança com o paciente e a família, bem como aperfeiçoar o processo de comunicação e LS com a criança, considerando suas particularidades.

A equipe de enfermagem constitui o principal grupo de profissionais no cuidado contínuo, ao realizar diferentes estratégias de atendimento às crianças hospitalizadas e seus cuidadores, por meio da educação em saúde^(3,5)^. Alguns fatores podem limitar a compreensão das informações relacionadas à saúde da criança por parte dos pais ou cuidadores, tais como nível de educação e escolaridade, baixo LS, ansiedade, preocupação com o sofrimento do filho, e comunicação inadequada ou ineficiente durante a internação^(6)^. As barreiras e habilidades comunicacionais limitadas dos profissionais de enfermagem podem interferir na capacidade de interpretar a comunicação verbal e não verbal do paciente pediátrico, além de dificultar a assimilação das informações compartilhadas pelos cuidadores sobre seu estado de saúde^(4)^.

A comunicação entre equipe de enfermagem e cuidadores deve promover uma relação de empatia, apoio e confiança, fundamental para o estabelecimento de vínculo entre os sujeitos envolvidos. Além disso, é uma ferramenta para reduzir o medo e a ansiedade de pacientes pediátricos e cuidadores, tornando o atendimento de emergência menos traumático^(7)^. Nessa perspectiva, contribui enquanto ferramenta de cuidado para o LS, que surge como construto intermediador das atividades educativas nos serviços de saúde^(1)^. O modelo 6D (seis dimensões) do LS para crianças e adolescentes envolve particularidades relacionadas ao desenvolvimento infantil e determinantes sociais da saúde. A apropriação de conhecimentos e ferramentas com foco no LS pode ampliar o protagonismo da criança no sentido de acessar, compreender, avaliar e aplicar informações a partir da interação com os profissionais de saúde^(8,9)^.

Estudos apontam a relevância e efetividade de intervenções com elementos que visam ampliar a empatia e a comunicação entre profissionais de saúde e crianças. Para atingir os objetivos, há necessidade de treinamento em estratégias de comunicação que qualifiquem a equipe de enfermagem a promover o LS de crianças e seus cuidadores^(4,7)^. A utilização de ferramentas com foco no LS desde a mais tenra idade é considerada um investimento promissor na saúde e bem-estar desses pacientes, tanto agora quanto ao longo de sua adolescência e vida adulta^(8)^.

Diante das concepções da comunicação em saúde como atividade para melhorar o LS aqui apresentadas, torna-se crucial o investimento em ações formativas de capacitação para que as instituições de saúde sejam responsivas ao LS^(10)^. Em vista disso, a educação permanente em saúde é um espaço de diálogo e problematização do cotidiano, desde que os profissionais sejam incitados a repensarem sua atuação em um processo permanente de qualificação. Esse movimento deve promover mudanças que melhorem a qualidade e a eficácia da atenção à saúde da população^(5)^.

Com base no potencial da educação permanente para mudanças na prática profissional e no processo de reflexão sobre a prática, este estudo teve como objetivo analisar os conhecimentos e perspectivas da equipe de enfermagem sobre um processo educativo com foco na comunicação e LS no contexto de uma emergência pediátrica.

## MÉTODO

### Desenho do Estudo

Trata-se de estudo qualitativo com referencial metodológico na pesquisa participativa em saúde, modalidade baseada na inter-relação entre os atores e saberes envolvidos em uma prática social e dialógica na perspectiva de Paulo Freire^(11)^, que permite incluir as vivências, experiências e reflexões dos participantes^(12)^. A presente pesquisa participativa foi realizada a partir de um processo educativo sobre comunicação e LS com a equipe de enfermagem de uma emergência pediátrica. Este artigo seguiu as diretrizes propostas pelo guia *COnsolidated criteria for REporting Qualitative research* (COREQ)^(13)^.

A pesquisa foi realizada durante o contexto da pandemia de COVID-19, respeitando os protocolos vigentes, como o distanciamento dos participantes na sala e o uso de máscaras.

### População e Amostra

O tamanho da amostra foi definido de forma intencional a partir de todos os participantes elegíveis, convidados por meio de visitas presenciais realizadas pela pesquisadora e via *WhatsApp* ^®^ e *Instagram* ^®^. Todos os interessados foram por ela informados de que se tratava de pesquisa baseada em um processo educativo realizado na emergência pediátrica, incluindo atividades síncronas e assíncronas, com carga horária total de 40 horas e entrega de certificado de participação após sua conclusão. No recrutamento inicial, 44 profissionais de enfermagem aceitaram participar, sendo dez enfermeiros e 34 técnicos de enfermagem. Contudo, seis técnicos de enfermagem não compareceram a nenhum encontro devido a motivos pessoais e indisponibilidade em envolver-se nas atividades propostas. Assim, a amostra final foi composta por 38 profissionais, sendo dez enfermeiros e 28 técnicos de enfermagem.

### Critérios de Seleção

Os critérios de inclusão consideraram profissionais de enfermagem da emergência pediátrica em plantão diurno, que desenvolviam ações de cuidado direto às crianças de 0 a 13 anos, 11 meses e 29 dias, com pelo menos um ano de experiência na função e disponibilidade para participar do processo educativo e de todas as atividades propostas durante o turno de trabalho. Os critérios de exclusão foram os profissionais que estavam de licença durante o período de realização da ação educativa.

### Cenário do Estudo

O processo educativo foi realizado em um hospital público brasileiro na cidade do Recife, PE, referência no atendimento pediátrico de urgência e emergência para crianças de 0 a 13 anos, 11 meses e 29 dias, com agravos de média e alta complexidade. O hospital recebe casos referenciados e regulados pela rede pública de saúde do estado de Pernambuco.

### Descrição do Processo Educativo

O planejamento do processo educativo foi elaborado com a intenção de agregar preceitos da ação-reflexão-ação presentes na Política Nacional de Educação Permanente em Saúde^(3)^. As estratégias de ensino-aprendizagem envolveram metodologias ativas, com ênfase no referencial teórico da aprendizagem experiencial desenvolvido por David Kolb^(14)^.

Para garantir maior viabilidade de participação dos profissionais, o processo educativo foi realizado durante o turno laboral diurno, nos horários de 10:00 às 11:30 (manhã) e 13:00 às 14:30 (tarde). Para atender a todos os participantes, os encontros foram repetidos três vezes, antes e durante os intervalos de trabalho, ajustando-se à dinâmica do serviço.

Após a inscrição e a assinatura do Termo de Consentimento Livre e Esclarecido (TCLE), os participantes integraram todas as atividades do processo educativo. No primeiro encontro, foram apresentados dois questionamentos: 1) Qual é a sua expectativa para esta formação sobre LS e comunicação com a criança? 2) Quais motivos levaram você a optar por participar deste processo educativo?

O processo educativo foi estruturado em quatro encontros, cada um com um tema específico: Tema 1: Resgatando a criança interior dos profissionais de enfermagem; Tema 2: Tipos de abordagens de comunicação com a criança/família em um ambiente hospitalar; Tema 3: Comunicação e letramento em saúde na prática profissional; Tema 4: Percepções sobre a implementação das abordagens comunicativas para a promoção do letramento em saúde no ambiente de trabalho (Quadro 1).

**Quadro 1:** Temas e estratégias educativas utilizadas nas sessões grupais – Recife, PE, Brasil, 2021.

Sessões grupais	Processo educativo
TEMA 1 – Resgatando a criança interior dos profissionais de enfermagem	**Dinâmica de sensibilização sobre a temática:** desenhos que marcaram a sua infância com idade 6-12 anos. Quem é essa criança interior? Como essa criança interior tem se comunicado com as crianças que estão sob meus cuidados na emergência? **Exposição dialogada:** conhecer os aspectos físicos, emocionais e sociais da criança. Imagens e pressupostos do livro “O Cérebro da Criança” (Daniel Siegel e Tina Payne).
TEMA 2 - Tipos de abordagens de comunicação com a criança/família em um ambiente hospitalar	**Exposição dialogada:** a partir da revisão de literatura sobre os tipos de abordagem de comunicação com a criança/família no contexto de hospitalização. Exemplificar as estratégias de comunicação com as abordagens já utilizadas no serviço. **Convidado:** Mirelly Barros (mestranda do Programa de Pós-Graduação em Saúde da Criança e do Adolescente da Universidade Federal de Pernambuco). Participação via plataforma *Google Meet* ^®^ com a temática “Contação de histórias como estratégia de comunicação com a criança no contexto de hospitalização”. Narração da história “O Coração e a Garrafa” (Oliver Jeffers).
TEMA 3 - Comunicação e letramento em saúde na prática profissional	**Apresentar os conceitos de comunicação e LS.** *ROLE PLAYING* **- Simulação construída a partir da Teoria do Aprendizado Experimental de David Kolb. Aplicação prática (dupla ou trio):** os participantes criaram duas situações envolvendo a equipe de enfermagem, com o propósito de articular as dimensões do LS e abordagens de comunicação. Nessas atividades, foram utilizadas estratégias de comunicação empática e promoção do LS.
TEMA 4 - Percepções sobre as abordagens comunicativas implementadas para a promoção do letramento em saúde no ambiente de trabalho	**Resgatar as atividades de ensino-aprendizagem no ambiente de trabalho:** aplicação prática de uma das estratégias de comunicação e articulação com o conceito de LS (filmagem de três a cinco minutos e autorreflexão).
Avaliação e autoavaliação dos participantes.	**Dinâmica “Árvore do conhecimento”:** para explorar as impressões dos participantes sobre a aplicação das abordagens comunicativas na promoção do LS na emergência pediátrica.

Fonte: elaborado pelo autor.

Cada encontro teve a seguinte sequência pedagógica: 1) dinâmica vivencial para identificação de conhecimentos e experiências prévias sobre o tema; 2) abordagem do tema principal pelo moderador, introduzindo novos conhecimentos; 3) perguntas de autorreflexão compartilhadas no grupo; 4) sugestões de atividades práticas para o ambiente de trabalho, encorajando os participantes a trazerem *feedbacks* para o encontro subsequente.

Ao final de cada encontro, uma pergunta era realizada para avaliar as informações sobre a compreensão e percepção dos participantes a partir de cada tema.

### Coleta dos Dados

Para a coleta dos dados, foi utilizada a técnica de grupos operativos (GOs), que coloca os participantes no centro do processo de aprendizagem, tornando-os protagonistas na construção do conhecimento^(15,16)^. As sessões grupais ocorreram entre outubro e novembro de 2021, organizadas da seguinte forma: abertura da sessão; apresentação dos participantes entre si; esclarecimento da dinâmica de discussão participativa; estabelecimento do *setting*; debate; síntese; e encerramento da sessão.

Os instrumentos de coleta de dados consistiram nas gravações das interações em grupo. Tais comunicações, mediadas pelas perguntas condutoras registradas na seção de descrição do processo educativo, foram o *corpus* principal de análise deste trabalho.

Ao todo, foram realizadas 12 sessões de GO com duração de cerca de uma hora e meia cada e presença de quatro a dez participantes, considerando uma média de oito a dez profissionais^(15)^. Os GOs foram conduzidos por um moderador estudante de pós-graduação, com treinamento teórico-prático em pesquisa qualitativa, e dois a três estudantes de graduação, os quais receberam um treinamento prévio para a condução e apoio às atividades.

O moderador foi responsável pelo preparo, organização e instrumentalização em todas as etapas, enquanto os observadores acompanharam as sessões e registraram os depoimentos dos participantes em notas de campo, além de auxiliar no manuseio dos equipamentos de gravação de áudio e imagem. O total de membros assistentes foi de nove observadores.

O local escolhido para a realização dos GO foi a sala de aula do próprio hospital, que oferecia estrutura, iluminação e temperatura adequadas, proporcionando um ambiente privativo e acolhedor. Os assentos foram dispostos em semicírculo, garantindo que a equipe de enfermagem, os moderadores e os observadores compartilhassem o mesmo campo de visão. Devido à pandemia, medidas sanitárias foram adotadas para garantir a segurança e o bem-estar dos pesquisadores e do público-alvo.

### Análise e Tratamento dos Dados

As narrativas foram gravadas em áudio e vídeo, com o consentimento dos participantes. As pausas discursivas e entonações captadas pelo moderador e observadores, bem como as expressões faciais, gestos e outras linguagens corporais, foram registrados em “notas de campo”. Ao final do processo educativo, os dados foram transcritos integralmente e submetidos à leitura e validação dos participantes, que, se necessário, poderiam fazer alterações.

O *corpus* analisado baseou-se nas narrativas produzidas ao longo do processo educativo e nas notas de campo, examinadas mediante codificação qualitativa, seguindo as etapas propostas por Graham Gibbs: codificação linha por linha; categorização dos temas; e análise dos dados^(17)^. Para auxiliar no processo de codificação e categorização temática, foi utilizado o *software* Atlas.ti (versão 8.0), que permitiu a segmentação do material coletado em códigos descritivos, grupos de códigos e categorias finais. A codificação foi realizada pela primeira autora (AKFS) em conjunto com a orientadora (MWLCM), e a síntese dos dados foi articulada com o referencial do LS.

### Aspectos Éticos

O estudo seguiu todas as diretrizes e prerrogativas legais estabelecidas pelas Resoluções n^o^ 466/2012 e n^o^ 510/2016 do Conselho Nacional de Saúde, e obteve aprovação do Comitê de Ética em Pesquisa da Universidade Federal de Pernambuco, conforme Parecer nº 4.755.034. Todos os participantes receberam orientações e esclarecimentos sobre o estudo e, em seguida, foi-lhes entregue o TCLE, devidamente assinado após o aceite para participar da pesquisa. Foram garantidos o sigilo e o anonimato dos participantes, identificados por meio de um sistema alfanumérico composto pelas letras “E” (Enfermeiros) e “T” (Técnicos de Enfermagem) e o número correspondente à ordem em que os participantes preencheram o TCLE.

## RESULTADOS

Os resultados foram obtidos de 38 profissionais, sendo dez enfermeiros e 28 técnicos de enfermagem, a maioria do sexo feminino (95,46%), com idade entre 26 e 62 anos. Esses profissionais tinham, em média, 17 anos de formação e cinco anos de atuação na instituição. Quanto à titulação, três enfermeiros (30%) e três técnicos de enfermagem (10,7%) possuíam especialização na área de atuação, e quatro enfermeiros (40%) eram mestres. Ao final do processo educativo, seis enfermeiros e 18 técnicos de enfermagem concluíram 70% das atividades propostas, totalizando assiduidade de 24 participantes.

A partir da codificação do *corpus*, foram sistematizadas duas categorias temáticas: 1 - Percepções e estratégias de comunicação da equipe de enfermagem; 2 - Conhecimentos e perspectivas do processo educativo fundamentados na comunicação e habilidades de letramento em saúde.

### Categoria 1- Percepções e Estratégias de Comunicação da Equipe de Enfermagem

#### Barreiras na Comunicação com a Criança

Em suas falas, os participantes citaram barreiras na comunicação com a criança, incluindo a necessidade de compreender aspectos físicos, emocionais e o nível de desenvolvimento infantil. O ambiente da emergência pediátrica, o tempo limitado para o cuidado e o letramento dos cuidadores também dificultavam a comunicação. Além disso, percebe-se que a gravidade da condição da criança influenciava a abordagem comunicativa em um cenário de emergência.

*Sabemos que é uma emergência pediátrica (....) principalmente na sala de estabilização, você não consegue ter um tempo maior para acalmar, para organizar, para trocar aquele olhar de empatia com a criança (...) diferente da ala verde e da ala amarela que você consegue, que não é uma criança de urgência, que não tá em uma situação de emergência, e aí você consegue ter um tempo melhor com aquela criança, apesar da demanda. Eu visualizei essas barreiras: serviço, o cenário do serviço, a criança dependendo da fase da doença em que ela se encontra e o nível de escolaridade dos pais.* (E02)

Os participantes identificaram na criança sentimentos como o medo do desconhecido, e expressaram inconformismo com a prática frequente de “segurar a criança à força” durante os procedimentos. Essa conduta foi vista como um obstáculo à relação de confiança e à comunicação entre a equipe de enfermagem e o binômio.

*A princípio, a criança tem muito medo! Só em ouvir (a palavra - acréscimo do autor) hospital assusta, aí depende muito da mãe ou do acompanhante. É importante abordar essa criança e conversar com ela; depende da idade da criança e do contexto, mas o medo é mesmo uma barreira (...).* (T17)

*Faz 4 anos que eu estou aqui, e não aceito isso de alguns bucos (cirurgiões bucomaxilofaciais - acréscimo do termo pelo autor), ortopedistas, médicos terem que suturar a criança segurando à força, sabe? Eu acho que isso causa um trauma desnecessário à criança, então a comunicação que fica para aquela criança é de que o profissional de saúde é uma pessoa ruim, malvada; ele é perverso, ele que segura a força e machuca.* (E07)

#### Estratégias Comunicativas na Relação com a Criança

Estratégias destacadas pelos participantes em suas experiências com as crianças incluíram o uso de linguagem lúdica, desenhos animados e uniformes personalizados com motivos infantis, visando à distração e interação durante procedimentos como troca de curativos, punção venosa e lavagem retal.

*Foi uma criança que iria fazer uma lavagem retal, e quem ia era outra técnica. Mas chamou tanta atenção a blusa de T12, da Frozen, que a criança disse: “Eu quero a Frozen!”. Então, é assim, querendo ou não, isso aproxima a criança e quebra o aspecto do medo.* (E06)

*Aí eu já fui tentando conversar com ele. Ele estava com a camisa do homem aranha, aí a gente já pega aquilo ali e vai tentando puxar um assunto que talvez seja do interesse dele, porque, às vezes, é uma música, um personagem que está na roupa (...) então, é uma forma da gente quebrar o gelo e começar a comunicação ali com aquela criança (...).* (E07)

*Eu disse: “É uma furadinha bem suportável”, assim, que nem uma formiguinha que a gente sente e não chora. Pronto, é a mesma coisa essa furada, aí ele disse: “A senhora pode me mostrar o tamanho dessa agulha?” (...) aí eu mostrei o tamanho do jelco, o amarelinho, 24. Veja o que foi que aconteceu na mente dele: “Ô tia, qual é o tamanho dos bebês daqui? Porque ele disse que a agulha era grande”.* (T07)

O brinquedo terapêutico foi citado como uma estratégia de comunicação com as crianças. Para os participantes, a interlocução efetiva envolve acolhimento e desenvolvimento de confiança, permitindo o acesso às informações de saúde durante as ações de cuidado.

*Mas acho que tem uma coisa chamada “brinquedo terapêutico” que a gente sempre tenta explicar para a criança o que vai ser feito nela, e serve para qualquer procedimento; isso alivia muito a tensão. Sendo que necessita de algum material para isso: bonequinho, material para simular a sonda e a seringa (...) para se aproximar, tocar e ter um momento com a criança (...).* (E14)

#### Estratégias Comunicativas com a Família

Quanto às estratégias de comunicação com a família, os participantes enfatizaram a importância de um acolhimento mais empático, como o uso da comunicação não verbal, em que expressões faciais e linguagem corporal atuam como facilitadores ou dificultadores dessa acolhida durante a internação da criança.

*Acho que a gente tem que ter muita paciência, saber falar (...). Você pode dar um não de diversas formas, você pode dar um não grosseiramente ou você pode dar um não delicadamente (...), a forma que você fala o tom que você usa. Porque muitas das vezes o corpo fala, o corpo com certeza fala! Suas expressões faciais e a movimentação do seu corpo, então (...).* (E08)

Os participantes percebiam que as questões socioeconômicas e o nível de escolaridade dos cuidadores/famílias que acompanham as crianças no pronto-socorro interferiam na compreensão, avaliação e aplicação das informações de saúde. Além disso, reconheciam a falta de ferramentas para usar uma comunicação mais simples e acessível.

*O primeiro ponto, eu acredito que é o nível de escolaridade dos pais, e eu percebo que quanto menor o nível de escolaridade, mais insegurança gera nesse processo de conhecimento da doença do filho e é mais difícil de conversar com essa pessoa, porque a gente não tem nenhuma preparação depois que você se forma para deixar de falar termos técnicos (...). Não é regredir, mas falar de uma forma que a população entenda.* (E02)

*Eu estou com aquela mãe ali daquele paciente, e eu vejo que ela não tem nenhuma qualidade, assim, de entendimento, e eu me doar até que eles possam entender o que eu estou falando. Me doar com compaixão. Me colocando ali naquele lugar.* (T07)

### Categoria 2 - Conhecimentos e Perspectivas do Processo Educativo Fundamentados na Comunicação e Habilidades de Letramento em Saúde

#### Acesso as Informações de Saúde

Os participantes destacaram que, além de compreender os aspectos físicos e emocionais da criança, é importante envolvêla no processo de cuidado, garantindo-lhe acesso a informações claras e verdadeiras sobre os procedimentos e tratamentos. Ademais, enfatizaram que os profissionais devem fazer o mesmo em relação aos cuidadores, solicitando seu apoio.

*Porque, se vamos puncionar, a gente sabe que eles vão chorar, vai gritar, vai correr, mas antes de tudo, nós temos que conversar e explicar o que está fazendo, mesmo quando muitas vezes eles não querem prestar atenção, mas é sempre bom estar explicando. Pedir para a mãe auxiliar também é muito importante (...). A mãe tranquiliza a criança enquanto fazemos o nosso trabalho.* (T06)

#### Compreensão e Avaliação das Informações de Saúde

Os participantes aplicaram estratégias de comunicação aprendidas no processo educativo, como o uso de fantoches. Tal vivência permitiu identificar os sentimentos da criança e da mãe, além de destacar o potencial do recurso lúdico para reduzir a dor e o medo. Também reconheceram a importância do suporte entre colegas no trabalho em equipe.

*Na verdade, a gente quis pegar uma criança grande porque achou que ela não ia chorar (...) mas a menina gritava, gritava muito, porque estava com muita dor abdominal (...)a gente tentou de alguma forma acalmar ela, e levou os fantoches para tirar a atenção dela (...). A mãe era muito ansiosa, porque ela não queria que a criança fosse para a cirurgia, e a gente de alguma forma tentou tranquilizar a mãe, medicou a menina, enfim, quando a gente passou o plantão, a menina estava dormindo bem tranquila (...).* (T14)

*(...) eu posso chegar para ela (a criança - acréscimo do autor) e explicar, tudo do começo, como vai ser aquela abordagem, para tranquilizá-la, trazer a família também como o ajudador nesse processo, porque cada vez que a gente se aproxima para fazer uma medicação, um procedimento, aquela criança entendendo a minha comunicação com ela, ela não vai ter medo (...).* (E01)

Outra estratégia utilizada pelos participantes foi a técnica *teach-back*, aplicada para explicar e reforçar com os familiares as informações de saúde e os tratamentos direcionados à criança na emergência pediátrica.

*Quando eu estava no curso, fui me aprimorar mais sobre o assunto, e eu vi que a técnica teach-back é a que finaliza tudo em termos de comunicação em saúde. Vi que ela é importantíssima porque gera benefício a médio e a longo prazo. Essa abordagem é o elo principal com o acompanhante, porque precisamos muito da ajuda dele.* (T02)

*Comunicação, eu acho que, no caso do letramento, é o ato de se fazer compreendido, porque não adianta eu falar e quem está recebendo a informação não compreender a minha fala. Então, houve aquela barreira, houve aquele filtro, ele não conseguiu compreender.* (E08)

#### Aplicação das Informações de Saúde

Os participantes compartilharam suas impressões e contribuições para o processo educativo, destacando-se a ampliação da capacidade de compreender as dores e as necessidades das crianças e seus cuidadores. Também foi observada a aplicação prática das estratégias de comunicação no próprio cotidiano, levando-se em conta as perspectivas das crianças e suas famílias.

*O curso, pra mim, serviu para aprimorar mais minha comunicação, paciência e empatia com a mãe e a criança, mostrar as coisas mais simples pra eles, observar mais o contexto da vida deles, pois assim fica mais claro para eles e para mim, para ajudar no dia a dia de cuidados da criança.* (T19)

*Me ajudou muito a forma de abordar as famílias, e agora já sabemos de forma mais clara como fazer essa abordagem, a linguagem, a forma de falar (...) até onde a pessoa consegue compreender, de forma clara e objetiva.* (T06)

Um ponto de destaque foi a percepção sobre a necessidade de processos contínuos de educação permanente na prática profissional como recurso para promover a ampliação de conhecimentos, habilidades e reflexões.

*(...) esse tipo de curso não é para você participar uma vez no ano; isso é educação permanente, e quando a gente vem e esquece, a gente tem um novo treinamento, a gente resgata (...) essa questão da educação em saúde é importante em todos os níveis da assistência; isso é a segurança do paciente, então isso (o letramento - acréscimo do autor) não pode ser uma educação pontual, tem que ser uma educação contínua (...).* (E08)

## DISCUSSÃO

A partir do objetivo de analisar os conhecimentos e perspectivas da equipe de enfermagem sobre um processo educativo com foco na comunicação e LS no contexto de uma emergência pediátrica, foi identificado o reconhecimento, por parte dos profissionais de enfermagem, das barreiras que impediam a comunicação empática e sensível com as crianças e, consequentemente, a ampliação de um olhar mais sensível em relação às necessidades de crianças e famílias atendidas na emergência pediátrica. As atividades reflexivas, aliadas às atividades práticas, forneceram elementos para uma comunicação em saúde geradora de mais satisfação no mundo do trabalho que, adicionalmente, estimulava a cooperação de crianças e familiares como aliados no cuidado de enfermagem.

A comunicação é a base do LS, essencial para estabelecer relações de confiança entre as pessoas^(18)^. Apesar das incertezas e do estresse presentes na circunstância de prática, os participantes compartilharam seus conhecimentos e experiências, o que contribuiu para a reflexão e aprimoramento das habilidades de comunicação e LS no contexto de hospitalização.

O cenário de emergência pediátrica é frequentemente difícil e caótico, e pode representar uma barreira/filtro no processo comunicativo, como apontam estudos^(19)^. Sob esse aspecto, o “medo do desconhecido” demonstrado pelas crianças foi identificado como um dos principais elementos desfavoráveis à comunicação. Além disso, outros estudos confirmam que pacientes pediátricos podem temer os profissionais de saúde, associando sua presença a procedimentos dolorosos^(2,20)^.

Os relatos apontaram experiências na comunicação verbal e não verbal que prejudicam a qualidade do cuidado da criança, como a prática de segurá-la “à força” durante procedimentos na emergência pediátrica. Essa conduta, segundo os participantes, impacta negativamente a forma como ela enfrenta a hospitalização, já que reconhecem a importância de respeitar seus sentimentos, anseios e particularidades. Estudos destacam a relevância de dar visibilidade ao papel ativo da criança no LS, garantindo sua voz e compreendendo suas necessidades por meio de estratégias que promovam o LS^(21,22)^.

A equipe de enfermagem identificou, ainda, outras barreiras de comunicação, como os aspectos socioeconômicos, o nível de escolaridade e o estado físico e emocional dos cuidadores/famílias durante a internação. Pesquisas destacam que fatores demográficos, socioeconômicos e o nível de LS da família influenciam o estado e comportamento de saúde da criança, impactando suas habilidades de LS^(21,23)^.

A falta de comunicação e informações de saúde durante o cuidado com a criança pela equipe de enfermagem pode gerar conflitos e angústia nos cuidadores/familiares. A forma como esses lidam com o tratamento influencia a participação da criança, bem como sua compreensão das informações de saúde e a aplicação desses conceitos em seu autocuidado^(8,24)^.

A formação de vínculos entre a equipe de enfermagem e a família desde o início da internação da criança é essencial para amenizar o estresse causado pelos procedimentos e tratamentos. Para isso, o profissional de saúde deve combinar amplas competências técnicas com conhecimentos e habilidades nas áreas da comunicação em saúde, mediação de conflitos, resolução de problemas, *marketing* em saúde e criatividade. Além disso, é fundamental possuir competências sociais que promovam uma abordagem mais humana, holística e aprofundada do paciente, considerando seu contexto^(9)^.

Quanto às estratégias de comunicação com a criança, alguns participantes relataram o uso de uniformes com temas infantis e adoção de linguagem lúdica para demonstrar os materiais que seriam utilizados na troca de curativos e punção venosa. A comunicação baseada em comparação e distração, recorrendo a imagens de desenhos animados durante os procedimentos, possibilitou à equipe de enfermagem interagir com a criança, respeitando seu nível de desenvolvimento cognitivo e socioemocional^(25)^.

Nossa percepção das crianças e dos jovens está profundamente ligada à nossa visão subjetiva da infância e ao papel social atribuído a esse público nas interações cotidianas. Nesse contexto, promover o LS de crianças e famílias requer o aperfeiçoamento de habilidades de LS com foco em atributos pessoais, considerando três categorias centrais: cognitiva (compreensão e avaliação); comportamental/operacional (acesso e aplicação); e afetiva/conativa (motivação). Essas competências interagem com fatores sociais e ambientais, sendo desenvolvidas ao longo das diferentes fases da infância e da vida^(8,26,27)^.

Na perspectiva do LS, as necessidades emocionais e sociais da criança devem ser atendidas pela equipe de enfermagem por meio de estratégias que a tornem sujeito ativo de seu processo de cuidado^(18,28)^. Alguns profissionais destacaram o uso do brinquedo terapêutico - abordagem facilitadora que utiliza um brinquedo estruturado para minimizar a dor e a ansiedade - como recurso para explicar os procedimentos à criança, facilitando sua adaptação às experiências vivenciadas no ambiente hospitalar^(28)^.

Estudo sobre a percepção da equipe de enfermagem em relação aos benefícios das estratégias lúdicas revelou que brincadeiras, fantoches, contação de histórias, jogos, acessórios com motivos infantis e músicas podem favorecer a adaptação, participação e colaboração da criança durante os procedimentos hospitalares^(25)^. Os participantes vivenciaram e perceberam os conhecimentos e perspectivas do processo educativo sobre comunicação e LS durante as atividades de ensino-aprendizagem no ambiente de trabalho. Alguns utilizaram, como estratégia lúdica, fantoches para orientar a criança com diagnóstico de apendicite e sua família, explicando-lhes o preparo e a realização de um procedimento cirúrgico.

Pesquisa realizada no Brasil em 2022 confirma que o uso de estratégias lúdicas, como a improvisação e personalização de bonecas e fantoches, facilita a comunicação entre profissionais de enfermagem e crianças hospitalizadas^(16)^. Ao adotar uma linguagem própria da infância, o profissional promove a interação e a participação ativa da criança, permitindo que ela expresse suas preocupações, medos e pontos de vista. Essa abordagem contribui para reduzir seu sofrimento emocional e minimizar as relações hierárquicas entre crianças e adultos^(22,24)^.

Outra estratégia de comunicação e LS adotada pela equipe de enfermagem foi a técnica *teach-back*, cujo principal objetivo é explicar as informações de saúde aos cuidadores e familiares da criança e verificar sua compreensão do que foi transmitido. Esse método, baseado no reensino, pode melhorar a compreensão dos envolvidos e reduzir erros de medicação que levam a novas readmissões^(4,29)^. Aplicar o *teach-back* de forma habitual é uma alternativa eficaz para triagem com base no LS, fornecendo aos profissionais de saúde informações relevantes, cumulativas e atualizadas a esse respeito e permitindo uma compreensão mais aprofundada das necessidades de LS dos cuidadores/da família^(29)^.

Os relatos dos participantes evidenciaram que a comunicação é um elo essencial para promover as habilidades de LS (acessar, compreender, avaliar e aplicar informações de saúde) em crianças hospitalizadas e seus cuidadores. Pesquisa realizada nos Estados Unidos em 2019 destaca que o LS transcende as habilidades individuais, estando fortemente associado ao modo como as informações e os cuidados são oferecidos pelos provedores de saúde^(4,30)^. Nesse sentido, os participantes observaram que o processo educativo sobre LS deve ser incorporado aos diversos níveis da assistência, ser contínuo e disponibilizado para todos os profissionais de saúde atuantes no serviço.

Portanto, adotar abordagens comunicativas que envolvam a criança em seu autocuidado - priorizando os aspectos físicos e socioemocionais da infância - permitirá aos profissionais de enfermagem desenvolverem uma comunicação simples, empática, acolhedora e humanizada nas ações de cuidado na emergência pediátrica. Logo, quanto mais simples e adequada for a comunicação dos profissionais de saúde com os cuidadores/família, mais fácil será sua compreensão e seu acesso às informações de saúde durante a recuperação e tratamento da criança^(1,24)^.

As limitações deste estudo incluem a avaliação imediata e a assiduidade dos participantes ao final do processo educativo, influenciadas pela escassez de pessoal, a natureza do serviço e a alta demanda de trabalho. Fatores externos interferentes em ambientes críticos de hospitalização e o cenário de pandemia com o novo coronavírus podem ter impactado o envolvimento e a participação dos profissionais de enfermagem nas atividades realizadas durante o processo educativo.

O engajamento dos profissionais em atividades de educação permanente, que demandam mais tempo para reflexão e construção de conhecimentos, parece ser um desafio para garantir maior validade externa dos dados. Outra limitação foi a análise da comunicação apenas sob a perspectiva dos profissionais de enfermagem, embora se reconheça a importância de avaliações centradas na criança e na família para avaliar a repercussão das ações de outros profissionais de saúde que compõem esse cenário.

## CONCLUSÃO

Esta investigação forneceu informações relevantes acerca dos conhecimentos e perspectivas dos profissionais de enfermagem sobre um processo educativo com foco na comunicação e LS no contexto de uma emergência pediátrica. Como observado, a comunicação dos profissionais de enfermagem pode influenciar a capacidade da criança/família de compreender, avaliar e aplicar as informações de saúde para a promoção do LS.

Além das estratégias lúdicas já utilizadas na emergência pediátrica, como desenhos animados, acessórios e uniformes com motivos infantis, os participantes ampliaram suas habilidades comunicativas baseadas no LS, como o reforço ao uso do brinquedo terapêutico, a técnica *teach-back* e os fantoches. Essas estratégias dialogam com as crianças e permitem motivá-las a participar das ações de cuidado.

As atividades de ensino-aprendizagem estimularam a participação ativa e reflexiva da equipe de enfermagem, com um repertório mais amplo de conhecimentos e habilidades de LS, especialmente no que se refere às estratégias e abordagens comunicativas específicas para as crianças. Além disso, potencializaram a relação de confiança entre os profissionais e a criança/família. Os dados apresentados podem subsidiar a aplicação de metodologias de ensino-aprendizagem em outros serviços de emergência pediátrica, como também em demais ambientes de cuidado nos quais a comunicação em saúde deve ser problematizada e ampliada por meio da reflexão sobre o trabalho.

## Data Availability

Os dados que dão suporte aos resultados deste estudo foram publicados na dissertação que originou o artigo, disponível em: https://repositorio.ufpe.br/handle/123456789/46079.

# Translation: Comunicación y alfabetización en salud en emergencia pediátrica: perspectivas del equipo de enfermería*

**DOI:** 10.1590/1980-220X-REEUSP-2024-0294es

**Authors:** Adélia Karla Falcão Soares, Francisca Márcia Pereira Linhares, Mirelly da Silva Barros, Fernanda Machado Silva-Rodrigues, Erika Acioli Gomes Pimenta, Queliane Gomes da Silva Carvalho, Maria Wanderleya Lavor Coriolano-Marinus

**Affiliations:** 1 Universidade Federal de Pernambuco, Programa de Pós-Graduação em Enfermagem, Recife, PE, Brasil.; 2 Universidade Federal de Pernambuco, Programa de Pós-Graduação em Saúde da Criança e Adolescente, Recife, PE, Brasil.; 3 Universidade de São Paulo, Escola de Enfermagem, São Paulo, SP, Brasil.; 4 Universidade Federal da Paraíba, Departamento de Enfermagem, João Pessoa, PB, Brasil.; 5 Universidade Federal de Pernambuco, Departamento de Enfermagem, Recife, PE, Brasil.

**Keywords:** Educación en Salud, Comunicación en Salud, Alfabetización en Salud, Niño Hospitalizado, Urgencias Médicas

## Abstract

**Objetivo:**

Analizar los conocimientos y perspectivas del equipo de enfermería sobre un proceso educativo centrado en la comunicación y la alfabetización en salud en el contexto de una emergencia pediátrica.

**Método:**

Estudio cualitativo, basado en la investigación participativa en salud y el marco teórico de la alfabetización en salud. El proceso educativo se realizó en una emergencia pediátrica entre octubre y noviembre de 2021, con la participación de diez enfermeros y 28 técnicos de enfermería. Los datos fueron recolectados de las interacciones y producciones de los grupos operativos durante el proceso educativo y analizados mediante codificación descriptiva y analítica, con apoyo del software Atlas.ti, versión 8.0.

**Resultados:**

Los participantes reconocieron las barreras en la comunicación con los niños y sus familias, ampliando su repertorio de estrategias comunicativas, reforzando el uso de juguetes terapéuticos, la técnica del teach-back y los títeres.

**Conclusión:**

Las actividades de enseñanza-aprendizaje estimularon la participación activa y reflexiva del equipo de enfermería, ampliando conocimientos y habilidades de alfabetización en salud.

## INTRODUCCIÓN

La alfabetización en salud (AS) es definida por la Organización Mundial de la Salud como la «capacidad de obtener, procesar y comprender información sobre la salud con el fin de tomar decisiones adecuadas para la gestión del autocuidado»^(1)^. Es un determinante social de la salud influido por condiciones estructurales, situacionales y contextuales que pueden limitar, favorecer o modificar la capacidad de los niños y adolescentes para actuar en contextos de salud. Este proceso está determinado por el entorno social en el que viven, incluidas las relaciones con cuidadores, amigos, profesores y profesionales sanitarios, entre otros^(2)^. La sala de urgencias pediátricas es un entorno de cuidados críticos que puede percibirse como impersonal, con restricciones en las actividades cotidianas del niño (como interactuar y explorar los diversos espacios que le rodean). Esto hace que la atención prestada por los profesionales sanitarios suponga un reto, ya que puede representar una amenaza momentánea o duradera para la integridad física y emocional del paciente pediátrico, especialmente cuando no se comprenden sus necesidades y no se utilizan estrategias específicas de atención integral^(3)^.

El personal de urgencias se enfrenta a traumatismos, enfermedades y fallecimientos que a menudo hacen que el escenario sea caótico, y establecer una buena comunicación puede resultar difícil^(4)^. Por lo tanto, además de los cuidados técnicos y emocionales, el profesional necesita establecer lazos de confianza con el paciente y la familia, así como perfeccionar la comunicación y el proceso de AS con el niño, considerando sus particularidades.

El equipo de enfermería es el principal grupo de profesionales en la continuidad de los cuidados, llevando a cabo diferentes estrategias de atención a los niños hospitalizados y a sus cuidadores a través de la educación en salud^(3,5)^. Algunos factores pueden limitar la comprensión de los padres o cuidadores de la información relacionada con la salud del niño, como el nivel de educación y escolarización, la baja AS, la ansiedad, la preocupación por el sufrimiento de su hijo y la comunicación inadecuada o ineficaz durante la hospitalización^(6)^. Las barreras y las limitadas habilidades comunicativas del personal de enfermería pueden interferir en su capacidad para interpretar la comunicación verbal y no verbal de los pacientes pediátricos, además de dificultarles la asimilación de la información compartida por los cuidadores sobre su estado de salud^(4)^.

La comunicación entre el personal de enfermería y los cuidadores debe promover una relación de empatía, apoyo y confianza, fundamental para establecer un vínculo entre los sujetos implicados. También es una herramienta para reducir el miedo y la ansiedad de los pacientes pediátricos y sus cuidadores, haciendo que los cuidados de urgencia sean menos traumáticos^(7)^. Desde esta perspectiva, contribuye como herramienta asistencial a la AS, que surge como constructo intermediario de las actividades educativas en los servicios de salud^(1)^. El modelo 6D (seis dimensiones) de AS para niños y adolescentes involucra particularidades relacionadas con el desarrollo infantil y los determinantes sociales de la salud. La apropiación de conocimientos y herramientas centrados en la AS puede aumentar el papel del niño en el acceso, comprensión, evaluación y aplicación de la información a través de la interacción con los profesionales de la salud^(8,9)^. Los estudios señalan la relevancia y la eficacia de las intervenciones con elementos dirigidos a aumentar la empatía y la comunicación entre los profesionales de la salud y los niños. Para la consecución de los objetivos, es necesaria la formación en estrategias de comunicación que capaciten al equipo de enfermería para promover la AS de los niños y sus cuidadores^(4,7)^. El uso de herramientas centradas en la AS desde edades tempranas se considera una inversión prometedora para la salud y el bienestar de estos pacientes, tanto ahora como a lo largo de su adolescencia y edad adulta^(8)^.

Dadas las concepciones de la comunicación en salud como actividad para mejorar la AS aquí presentadas, es crucial invertir en actividades de formación para que las instituciones sanitarias sean receptivas a la AS^(10)^. Frente a esto, la educación en salud permanente es un espacio de diálogo y problematización de la vida cotidiana, siempre que se estimule a los profesionales a repensar su trabajo en un proceso permanente de cualificación. Ese movimiento debe promover cambios que mejoren la calidad y la eficacia de la atención a la salud de la población^(5)^.

Partiendo del potencial de la formación continuada para cambios en la práctica profesional y del proceso de reflexión sobre la práctica, este estudio tuvo como objetivo analizar los conocimientos y perspectivas del equipo de enfermería sobre un proceso educativo centrado en la comunicación y AS en el contexto de una urgencia pediátrica.

## MÉTODO

### Diseño del Estudio

Se trata de un estudio cualitativo con referencia metodológica en la investigación participativa en salud, modalidad basada en la interrelación entre los actores y saberes implicados en una práctica social y dialógica desde la perspectiva de Paulo Freire^(11)^, que permite la inclusión de las experiencias y reflexiones de los participantes^(12)^. Esta investigación participativa se llevó a cabo a través de un proceso educativo sobre comunicación y AS con el personal de enfermería de un servicio de urgencias pediátricas. Este artículo siguió las pautas propuestas por la guía *COnsolidated criteria for REporting Qualitative research* (COREQ)^(13)^.

La investigación se realizó en el contexto de la pandemia de COVID-19, respetando los protocolos vigentes, como el distanciamiento de los participantes en la sala y el uso de mascarillas.

### Población y Muestra

El tamaño de la muestra se definió intencionalmente en base a todos los participantes elegibles, que fueron invitados a través de visitas presenciales del investigador y vía *WhatsApp* ^®^ e *Instagram* ^®^. Todos los interesados fueron informados por la investigadora de que la investigación se basaba en un proceso educativo realizado en los servicios de urgencias pediátricas, que incluía actividades síncronas y asíncronas, con una carga de trabajo total de 40 horas y la obtención de un certificado de participación al finalizar. Durante el reclutamiento inicial, 44 profesionales de enfermería aceptaron participar, diez de ellos enfermeras y 34 técnicos de enfermería. Sin embargo, seis técnicos de enfermería no asistieron a ninguna de las reuniones por motivos personales y por no querer implicarse en las actividades propuestas. Por lo tanto, la muestra final estaba formada por 38 profesionales, diez de ellos enfermeros y 28 técnicos de enfermería.

### Criterios de Selección

Los criterios de inclusión fueron profesionales de enfermería de urgencias pediátricas de guardia diurna, que prestaran atención directa a niños de 0-13 años, 11 meses y 29 días, con al menos un año de experiencia en la función y dispuestos a participar en el proceso educativo y en todas las actividades propuestas durante su turno. Los criterios de exclusión fueron los profesionales que estuvieran de baja durante el periodo de la actividad educativa.

### Escenario del Estudio

El proceso educativo se realizó en un hospital público brasileño de la ciudad de Recife, PE, referencia en la atención pediátrica de urgencia y emergencia para niños de 0 a 13 años, 11 meses y 29 días, con patologías de mediana y alta complejidad. El hospital recibe casos referidos y regulados por la red pública de salud del estado de Pernambuco.

### Descripción del Proceso Educativo

La planificación del proceso educativo fue diseñada con la intención de agregar los preceptos de acción-reflexión-acción presentes en la Política Nacional de Educación Permanente en salud^(3)^. Las estrategias de enseñanza-aprendizaje involucraron metodologías activas, con énfasis en el marco teórico del aprendizaje experiencial desarrollado por David Kolb^(14)^.

Para garantizar una mayor viabilidad de participación de los profesionales, el proceso educativo se realizó en horario diurno, de 10h a 11h30 (mañana) y de 13h a 14h30 (tarde). Para atender a todos los participantes, las reuniones se repitieron tres veces, antes y durante las pausas de trabajo, ajustándose a la dinámica del servicio.

Tras inscribirse y firmar el Formulario de Consentimiento Informado (FCI), los participantes tomaron parte en todas las actividades del proceso educativo. En la primera reunión, se formularon dos preguntas: 1) ¿Cuáles son sus expectativas para esta formación sobre AS y comunicación con niños? 2) ¿Qué razones le llevaron a elegir participar en este proceso educativo?

El proceso formativo se estructuró en cuatro encuentros, cada uno con un tema específico: Tema 1: Rescatando el niño interior de los profesionales de enfermería; Tema 2: Tipos de abordajes comunicativos con el niño/familia en ambiente hospitalario; Tema 3: Comunicación y alfabetización en salud en la práctica profesional; Tema 4: Percepciones sobre la implementación de abordajes comunicativos para promover la alfabetización en salud en el ambiente de trabajo (Cuadro 1).

**Cuadro 1:** Temas y estrategias pedagógicas utilizadas en las sesiones de grupo – Recife, PE, Brasil, 2021.

Sesiones grupales	Proceso educativo
TEMA 1 - Rescatar al niño interior de los profesionales de enfermería	**Dinámica de sensibilización sobre el tema:** dibujos que marcaron tu infancia entre los 6 y los 12 años. ¿Quién es este niño interior? ¿Cómo se ha comunicado este niño interior con los niños a mi cargo en urgencias? **Diálogo**: conocer los aspectos físicos, emocionales y sociales del niño. Imágenes y supuestos del libro «El cerebro del niño» (Daniel Siegel y Tina Payne).
TEMA 2 - Tipos de enfoques de comunicación con el niño o la familia en un entorno hospitalario	**Presentación dialogada:** a partir de una revisión bibliográfica sobre los tipos de abordaje comunicativo con el niño/familia en el contexto de la hospitalización. Ejemplificar estrategias de comunicación con los abordajes ya utilizados en el servicio. **Invitada:** Mirelly Barros (estudiante de Maestría en el Programa de Postgrado en Salud del Niño y del Adolescente de la Universidad Federal de Pernambuco). Participación a través de la plataforma *Google Meet* ^®^ con el tema «Storytelling como estrategia de comunicación con los niños en el contexto de la hospitalización». Narración del cuento «El corazón y la botella» (Oliver Jeffers).
TEMA 3 - Comunicación en salud y alfabetización en la práctica profesional	**Introducción a los conceptos de comunicación y AS.** *ROLE PLAYING* - Simulación basada en la Teoría del Aprendizaje Experiencial de David Kolb. **Aplicación práctica (en pareja o trío):** los participantes crearon dos situaciones que implicaban al equipo de enfermería, con el objetivo de articular las dimensiones de los enfoques de AS y comunicación. En estas actividades, se utilizaron estrategias de comunicación empática y de promoción dla AS.
TEMA 4 - Percepciones de los enfoques comunicativos aplicados para promover la alfabetización en salud en el lugar de trabajo	**Recuperación de las actividades de enseñanza-aprendizaje en el lugar de trabajo:** aplicación práctica de una de las estrategias de comunicación y articulación con el concepto de AS (filmación de tres a cinco minutos y autorreflexión).
Evaluación y autoevaluación de los participantes.	**Dinámica del «árbol del conocimiento»:** explorar las impresiones de los participantes sobre la aplicación de enfoques comunicativos para promover la AS en las urgencias pediátricas.

Fuente: Autores

Cada reunión tuvo la siguiente secuencia pedagógica: 1) dinámica experiencial para identificar los conocimientos y experiencias previas sobre el tema; 2) planteamiento del tema principal por parte del moderador, introduciendo nuevos conocimientos; 3) preguntas de autorreflexión compartidas en el grupo; 4) sugerencias de actividades prácticas para el lugar de trabajo, animando a los participantes a traer *sus comentarios* a la siguiente reunión.

Al final de cada reunión, se formulaba una pregunta para evaluar la información sobre la comprensión y percepción de cada tema por parte de los participantes.

### Recolección de Datos

Los datos se recogieron utilizando la técnica del grupo operativo (GO), que sitúa a los participantes en el centro del proceso de aprendizaje, convirtiéndolos en protagonistas de la construcción del conocimiento^(15–16)^. Las sesiones de grupo tuvieron lugar entre octubre y noviembre de 2021 y se organizaron de la siguiente manera: apertura de la sesión; presentación de los participantes; clarificación de la dinámica de discusión participativa; establecimiento del *marco*; debate; síntesis; y cierre de la sesión.

Los instrumentos de recogida de datos consistieron en grabaciones de las interacciones del grupo. Estas comunicaciones, mediadas por las preguntas orientativas registradas en la sección que describe el proceso educativo, constituyeron el *corpus* principal para analizar este trabajo.

Se celebraron un total de 12 sesiones de GO, cada una de las cuales duró en torno a una hora y media y a la que asistieron entre cuatro y diez participantes, considerando una media de ocho a diez profesionales^(15)^. Los GO fueron dirigidas por un estudiante de postgrado moderador, con formación teórica y práctica en investigación cualitativa, y dos o tres estudiantes de grado, que recibieron formación previa para dirigir y apoyar las actividades.

El moderador se encargó de preparar, organizar e instrumentalizar todas las fases, mientras que los observadores siguieron las sesiones y registraron los testimonios de los participantes en notas de campo, además de ayudar a manejar el equipo de grabación de audio e imagen. En total hubo nueve observadores.

El lugar elegido para los GO fue el aula del propio hospital, que ofrecía una estructura, iluminación y temperatura adecuadas, proporcionando un entorno privado y acogedor. Los asientos se dispusieron en semicírculo, asegurando que el equipo de enfermería, los moderadores y los observadores compartieran el mismo campo de visión. Debido a la pandemia, se adoptaron medidas sanitarias para garantizar la seguridad y el bienestar de los investigadores y del público objetivo.

### Análisis y Tratamiento de los Datos

Las narraciones se grabaron en audio y vídeo con el consentimiento de los participantes. Las pausas discursivas y las entonaciones captadas por el moderador y los observadores, así como las expresiones faciales, los gestos y demás lenguaje corporal, se registraron en «notas de campo». Al final del proceso pedagógico, los datos se transcribieron íntegramente y se sometieron a la lectura y validación de los participantes, que podían introducir cambios si era necesario.

El *corpus* analizado se basó en las narraciones producidas durante el proceso educativo y las notas de campo, que se examinaron mediante codificación cualitativa, siguiendo las etapas propuestas por Graham Gibbs: codificación línea por línea; categorización de temas; y análisis de datos^(17)^. Para el proceso de codificación y categorización temática se utilizó *el programa* Atlas.ti (versión 8.0), que permitió segmentar el material Recolectado en códigos descriptivos, grupos de códigos y categorías finales. La codificación fue realizada por la primer autora (AKFS) junto con la supervisora (MWLCM), y los datos se resumieron utilizando el marco de la AS.

### Aspectos Éticos

El estudio siguió todas las directrices y prerrogativas legales establecidas por las Resoluciones n.^º^ 466/2012 y n.^º^ 510/2016 del Consejo Nacional de Salud, y fue aprobado por el Comité de Ética en Investigación de la Universidad Federal de Pernambuco, según el Dictamen n.º 4.755.034. Todos los participantes recibieron orientación y explicaciones sobre el estudio y luego se les entregó el FCI, debidamente firmado después de aceptar participar en la investigación. Se garantizó la confidencialidad y el anonimato de los participantes, que fueron identificados mediante un sistema alfanumérico compuesto por las letras «E» (Enfermeros) y «T» (Técnicos de Enfermería) y el número correspondiente al orden en que los participantes rellenaron el FCI.

## RESULTADOS

Los resultados se obtuvieron de 38 profesionales, de los cuales diez eran enfermeros y 28 técnicos de enfermería, la mayoría mujeres (95,46%), con edades comprendidas entre 26 y 62 años. Estos profesionales tenían una media de 17 años de formación y cinco años de experiencia en la institución. En cuanto a las cualificaciones, tres enfermeras (30%) y tres técnicos de enfermería (10,7%) tenían especializaciones en su campo, y cuatro enfermeras (40%) tenían másteres. Al final del proceso educativo, seis enfermeras y 18 técnicos de enfermería habían completado el 70% de las actividades propuestas, lo que supone un total de 24 participantes.

A partir de la codificación del *corpus*, se sistematizaron dos categorías temáticas: 1 - Percepciones y estrategias de comunicación del equipo de enfermería; 2 - Conocimientos y perspectivas del proceso educativo basado en habilidades de comunicación y alfabetización en salud.

### Categoría 1- Percepciones y Estrategias de Comunicación del Equipo de Enfermería

#### Barreras Para la Comunicación con los Niños

Los participantes mencionaron barreras para la comunicación con el niño, incluyendo la necesidad de comprender aspectos físicos y emocionales y el nivel de desarrollo del niño. El entorno de la urgencia pediátrica, el escaso tiempo de atención y la alfabetización de los cuidadores también dificultaban la comunicación. Además, la gravedad del estado del niño influyó en el enfoque comunicativo en un contexto de urgencia.

*Sabemos que es una urgencia pediátrica (....), sobre todo en la sala de estabilización, no tienes más tiempo para calmarte, para organizarte, para intercambiar esa mirada de empatía con el niño (...) a diferencia de la sala verde y la sala amarilla donde sí puedes, porque no es una situación de urgencia, y puedes pasar mejor tiempo con ese niño, a pesar de la demanda. Visualicé estas barreras: el servicio, el entorno del servicio, el niño dependiendo de la fase de la enfermedad en la que se encuentre y el nivel educativo de los padres.* (E02)

Los participantes identificaron sentimientos en el niño como el miedo a lo desconocido, y expresaron su descontento con la práctica frecuente de «sujetar al niño por la fuerza» durante los procedimientos. Este comportamiento se consideró un obstáculo para la relación de confianza y comunicación entre el equipo de enfermería y el binomio.

*Al principio, ¡el niño está muy asustado! Sólo con oír (la palabra - añadido del autor) se asusta en el hospital, así que depende mucho de la madre o del cuidador. Es importante acercarse al niño y hablarle; depende de la edad del niño y del contexto, pero el miedo es realmente una barrera (...).* (T17)

*Llevo aquí 4 años y no acepto que algunos cirujanos orales, cirujanos ortopédicos, médicos tengan que suturar al niño sujetándolo por la fuerza, ¿sabes? Yo creo que eso le causa un trauma innecesario al niño, entonces la comunicación que le queda a ese niño es que el profesional de salud es una persona mala, malvada; es perverso, es el que agarra al niño y lo lastima.»* (E07)

#### Estrategias Comunicativas en la Relación con el Niño

Entre las estrategias destacadas por los participantes en sus experiencias con niños, están el uso de lenguaje lúdico, dibujos animados y uniformes personalizados con motivos infantiles, con el objetivo de distraer e interactuar durante procedimientos como cambio de apósitos, venopunción y lavado rectal.*Era un niño el que iba a hacer un lavado rectal, y era otro técnico el que iba. Pero la blusa de Frozen de T12 llamó tanto la atención que el niño dijo: «¡Quiero Frozen!». Así que, te guste o no, esto acerca al niño y rompe el aspecto del miedo.* (E06)

*Entonces empecé a intentar hablar con él. Llevaba una camiseta de Spiderman, así que la cogimos e intentamos hablarle de algo que pudiera interesarle, porque a veces es una canción, un personaje que está en la ropa (...) así que es una forma de romper el hielo y empezar a comunicarnos con ese niño (...).* (E07)

*Le dije: «Es un pinchacito muy soportable», como una hormiguita que sientes y no lloras. Entonces me dijo: «¿Me enseñas el tamaño de esta aguja?» (...) Entonces le enseñé el tamaño de la aguja, la amarilla, la 24. Mira lo que pasó por su cabeza: «Tía, ¿cómo son de grandes los bebés de aquí? Porque dijo que la aguja era grande».* (T07)

El juguete terapéutico fue citado como estrategia de comunicación con los niños. Para los participantes, un diálogo eficaz implica acoger y desarrollar la confianza, permitiendo el acceso a la información sanitaria durante las actividades asistenciales.

*Pero creo que hay algo que se llama «juguete terapéutico», que siempre intentamos explicar al niño lo que se le va a hacer, y se puede utilizar para cualquier procedimiento; alivia mucho la tensión. Se necesita material para ello: un muñeco, material para simular la sonda y la jeringuilla (...) para acercarse, tocar y tener un momento con el niño (...).*(E14)

#### Estrategias de Comunicación con la Familia

En cuanto a las estrategias de comunicación con la familia, los participantes destacaron la importancia de una acogida más empática, como el uso de la comunicación no verbal, en la que las expresiones faciales y el lenguaje corporal actúan como facilitadores o dificultadores durante la estancia del niño en el hospital.

*Creo que hay que tener mucha paciencia, saber hablar (...). Se puede decir que no de diferentes maneras, se puede decir que no bruscamente o se puede decir que no con suavidad (...), la forma de hablar, el tono que se utiliza. Porque a menudo el cuerpo habla, ¡el cuerpo ciertamente habla! Tus expresiones faciales y la forma en que se mueve tu cuerpo, así que (...).* (E08)

Los participantes se dieron cuenta de que las cuestiones socioeconómicas y el nivel de educación de los cuidadores/familias que acompañan a los niños a urgencias interferían en la comprensión, evaluación y aplicación de la información sanitaria. Además, reconocieron la falta de herramientas para utilizar una comunicación más sencilla y accesible.

*El primer punto, creo, es el nivel de educación de los padres, y me doy cuenta de que cuanto más bajo es el nivel de educación, más inseguridad genera en este proceso de conocer la enfermedad de su hijo y es más difícil hablar con esta persona, porque no tenemos ninguna preparación después de graduarte para dejar de hablar en términos técnicos (...). No se trata de retroceder, sino de hablar de una manera que la gente entienda.* (E02)

*Estoy allí con la madre de esa paciente, y veo que no tiene ninguna cualidad de comprensión, y me entrego hasta que puedan entender lo que estoy diciendo. Me entrego con compasión. Poniéndome en ese lugar.* (T07)

### Categoría 2 - Conocimientos y Perspectivas del Proceso Educativo Basado en la Comunicación y la Alfabetización en Salud

#### Acceso a la Información en Salud

Los participantes destacaron que, además de comprender los aspectos físicos y emocionales del niño, es importante involucrarlos en el proceso de atención, garantizándoles el acceso a información clara y veraz sobre procedimientos y tratamientos. También destacaron que los profesionales deben hacer lo mismo con los cuidadores, solicitando su apoyo.

*Porque si vamos a pinchar, sabemos que van a llorar, gritar, correr, pero antes de nada tenemos que hablar y explicar lo que estamos haciendo, aunque muchas veces no quieran prestar atención, pero siempre es bueno explicar. Pedir ayuda a la madre también es muy importante (...). La madre tranquiliza al niño mientras nosotros hacemos nuestro trabajo.* (T06)

#### Comprensión y Evaluación de la Información en Salud

Los participantes aplicaron estrategias de comunicación aprendidas en el proceso educativo, como el uso de marionetas. Esta experiencia les permitió identificar los sentimientos del niño y de la madre, así como destacar el potencial de los recursos lúdicos para reducir el dolor y el miedo. También reconocieron la importancia del apoyo de los compañeros en el trabajo en equipo.

*En realidad, queríamos llevar a una niña grande porque pensábamos que no lloraría (...) pero la pequeña gritaba, gritaba mucho, porque tenía mucho dolor abdominal (...) intentamos calmarla de alguna manera, y cogimos las marionetas para desviar su atención (...). La madre estaba muy ansiosa, porque no quería que la niña entrase en el quirófano, e intentamos tranquilizar a la madre, medicamos a la niña, y cuando terminamos el turno, la niña dormía muy tranquila (...).* (T14)

*(...) Puedo llegar hasta ella (la niña - adición de la autora) y explicarle, desde el principio, cómo va a ser el abordaje, tranquilizarla, traer a la familia como ayudantes en este proceso, porque cada vez que nos acercamos a ella para hacer una medicación, un procedimiento, si esa niña entiende mi comunicación con ella, no va a tener miedo (...).* (E01)

Otra estrategia utilizada por los participantes fue la técnica *teach-back*, utilizada para explicar y reforzar con los familiares la información sanitaria y los tratamientos para niños en urgencias pediátricas.

*Cuando estuve en el curso, aprendí más sobre el tema, y vi que la técnica teach-back es lo que finaliza todo en términos de comunicación en salud. Me di cuenta de que es sumamente importante porque genera beneficios a medio y largo plazo. Este enfoque es el principal vínculo con el cuidador, porque realmente necesitamos su ayuda.* (T02)

*La comunicación, creo, en el caso de la alfabetización, es el acto de ser entendido, porque no tiene sentido que yo hable y la persona que recibe la información no entienda lo que estoy diciendo. Así que había esa barrera, había ese filtro, no podían entender”* (E08).

#### Aplicación de la Información en Salud

Los participantes compartieron sus impresiones y contribuciones al proceso educativo, destacando la mayor capacidad para comprender el dolor y las necesidades de los niños y sus cuidadores. También señalaron la aplicación práctica de estrategias de comunicación en su propia vida diaria, teniendo en cuenta las perspectivas de los niños y sus familias.

*Para mí, el curso ha servido para mejorar mi comunicación, paciencia y empatía con las madres y los niños, para mostrarles las cosas más sencillas, para observar más el contexto de sus vidas, porque esto hace que sea más claro para ellos y para mí, para ayudar en el cuidado diario del niño.* (T19)

*Me ha ayudado mucho la forma de abordar a las familias, ahora sabemos más claramente cómo abordarlas, el lenguaje, la forma de hablar (...) hasta donde la persona pueda entender, de forma clara y objetiva.* (T06)

Un aspecto destacado fue la toma de conciencia de la necesidad de procesos de formación continua en la práctica profesional como recurso para promover la ampliación de conocimientos, habilidades y reflexiones.

*(...) este tipo de curso no es para que uno asista una vez al año; esto es educación permanente, y cuando venimos y nos olvidamos, tenemos nueva formación, nos recuperamos (...) este tema de la educación en salud es importante en todos los niveles de atención; esto es seguridad del paciente, entonces esto (alfabetización - adición del autor) no puede ser una educación puntual, tiene que ser una educación continua (...).* (E08)

## DISCUSIÓN

Con el objetivo de analizar los conocimientos y perspectivas del equipo de enfermería sobre un proceso educativo centrado en la comunicación y AS en el contexto de una urgencia pediátrica, identificamos el reconocimiento por parte de los profesionales de enfermería de las barreras que impedían una comunicación empática y sensible con los niños y, consecuentemente, la ampliación de una visión más sensible de las necesidades de los niños y familias atendidas en urgencias pediátricas. Las actividades reflexivas, combinadas con las actividades prácticas, proporcionaron elementos para la comunicación en salud que generaron más satisfacción en el mundo del trabajo y, además, fomentaron la cooperación de los niños y las familias como aliados en los cuidados de enfermería.

La comunicación es la base de la AS, esencial para establecer relaciones de confianza entre las personas^(18)^. A pesar de las incertidumbres y el estrés presentes en el escenario de la práctica, los participantes compartieron sus conocimientos y experiencias, lo que contribuyó a la reflexión y a la mejora de las habilidades de comunicación y AS en el contexto de la hospitalización.

El entorno de las urgencias pediátricas es a menudo difícil y caótico, y puede representar una barrera/filtro en el proceso de comunicación, como han señalado algunos estudios^(19)^. En este sentido, el «miedo a lo desconocido» demostrado por los niños se identificó como uno de los principales elementos desfavorables para la comunicación. Además, otros estudios confirman que los pacientes pediátricos pueden temer a los profesionales sanitarios, asociando su presencia a procedimientos dolorosos^(2,20)^.

Los informes señalaron experiencias de comunicación verbal y no verbal que menoscaban la calidad de la atención a los niños, como la práctica de sujetarlos «por la fuerza» durante los procedimientos pediátricos de urgencia. Según los participantes, este comportamiento repercute negativamente en la forma en que afrontan la hospitalización, ya que reconocen la importancia de respetar sus sentimientos, deseos y particularidades. Los estudios destacan la importancia de dar visibilidad al papel activo del niño en la AS, garantizando su voz y comprendiendo sus necesidades a través de estrategias que promuevan la AS^(21,22)^.

El equipo de enfermería también identificó otras barreras de comunicación, como los aspectos socioeconómicos, el nivel de educación y el estado físico y emocional de los cuidadores/familiares durante la hospitalización. Las investigaciones destacan que los factores demográficos y socioeconómicos y el nivel de AS de la familia influyen en el estado de salud y el comportamiento del niño, lo que repercute en sus habilidades de AS^(21,23)^.

La falta de comunicación e información en salud durante el cuidado del niño por parte del equipo de enfermería puede provocar conflictos y angustia entre los cuidadores/familiares. La forma en que afrontan el tratamiento influye en la participación del niño, así como en su comprensión de la información sanitaria y en la aplicación de estos conceptos en su autocuidado^(8,24)^.

Establecer vínculos entre el equipo de enfermería y la familia desde el inicio de la hospitalización del niño es esencial para reducir el estrés causado por los procedimientos y tratamientos. Para ello, los profesionales de la salud deben combinar amplias competencias técnicas con conocimientos y habilidades en los ámbitos de la comunicación en salud, la mediación de conflictos, la resolución de problemas, el *marketing* sanitario y la creatividad. Además, es fundamental contar con habilidades sociales que promuevan un abordaje más humano, holístico y profundo del paciente, considerando su contexto^(9)^.

En cuanto a las estrategias para comunicarse con los niños, algunos participantes informaron de la utilización de uniformes con temas infantiles y el uso de un lenguaje lúdico para demostrar los materiales que se utilizarían para el cambio de apósitos y la venopunción. La comunicación basada en la comparación y la distracción, utilizando imágenes de dibujos animados durante los procedimientos, permitió al equipo de enfermería interactuar con el niño respetando su nivel de desarrollo cognitivo y socioemocional^(25)^.

Nuestra percepción de los niños y jóvenes está profundamente vinculada a nuestra visión subjetiva de la infancia y al papel social atribuido a este público en las interacciones cotidianas. En este contexto, promover la AS de los niños y las familias requiere mejorar las habilidades de AS centradas en atributos personales, considerando tres categorías centrales: cognitiva (comprensión y evaluación); conductual/operativa (acceso y aplicación); y afectiva/conativa (motivación). Estas competencias interactúan con factores sociales y ambientales y se desarrollan a lo largo de las diferentes etapas de la infancia y de la vida^(8,26,27)^.

Desde la perspectiva de la AS, las necesidades emocionales y sociales del niño deben ser atendidas por el equipo de enfermería a través de estrategias que lo conviertan en sujeto activo de su proceso de atención^(18,28)^. Algunos profesionales han destacado el uso del juguete terapéutico - abordaje facilitador que utiliza un juguete estructurado para minimizar el dolor y la ansiedad - como recurso para explicar los procedimientos al niño, facilitando su adaptación a las vivencias en el ambiente hospitalario^(28)^.

Un estudio sobre la percepción del equipo de enfermería de los beneficios de las estrategias lúdicas reveló que los juegos, las marionetas, los cuentos, los juegos, los accesorios con motivos infantiles y la música pueden favorecer la adaptación, la participación y la colaboración de los niños durante los procedimientos hospitalarios^(25)^. Los participantes experimentaron y se dieron cuenta de los conocimientos y perspectivas del proceso educativo sobre comunicación y AS durante las actividades de enseñanza-aprendizaje en el lugar de trabajo. Algunos utilizaron marionetas como estrategia lúdica para orientar a los niños diagnosticados de apendicitis y a sus familias, explicándoles cómo prepararse y llevar a cabo un procedimiento quirúrgico.

Una investigación realizada en Brasil en 2022 confirma que el uso de estrategias lúdicas, como la improvisación y la personalización de muñecos y marionetas, facilita la comunicación entre los profesionales de enfermería y los niños hospitalizados^(16)^. Al adoptar el lenguaje de la infancia, los profesionales promueven la interacción y la participación activa de los niños, permitiéndoles expresar sus preocupaciones, miedos y puntos de vista. Este enfoque contribuye a reducir su malestar emocional y a minimizar las relaciones jerárquicas entre niños y adultos^(22,24)^.

Otra estrategia de comunicación y AS adoptada por el equipo de enfermería fue la técnica *teach-back*, cuyo principal objetivo es explicar la información sanitaria a los cuidadores y a la familia del niño y comprobar que han entendido lo que se les ha transmitido. Este método, basado en la re-enseñanza, puede mejorar la comprensión de los implicados y reducir los errores de medicación que conducen a nuevos reingresos^(4,29)^. La aplicación *del teach-back* de forma regular es una alternativa eficaz al cribado basado en la AS, ya que proporciona a los profesionales sanitarios información relevante, acumulativa y actualizada sobre la AS y permite una comprensión más profunda de las necesidades dla AS de los cuidadores/familiares^(29)^.

Los informes de los participantes mostraron que la comunicación es un eslabón esencial en la promoción de las habilidades de AS (acceso, comprensión, evaluación y aplicación de la información sanitaria) en los niños hospitalizados y sus cuidadores. Las investigaciones llevadas a cabo en Estados Unidos en 2019 destacan que la AS trasciende las habilidades individuales y está fuertemente asociada a la forma en que la información y la atención son ofrecidas por los proveedores de atención sanitaria^(4,30)^. En este sentido, los participantes señalaron que el proceso educativo sobre AS debe incorporarse a los distintos niveles asistenciales, ser continuo y ponerse a disposición de todos los profesionales sanitarios que trabajan en el servicio.

Por tanto, la adopción de enfoques comunicativos que impliquen al niño en su autocuidado - priorizando los aspectos físicos y socioemocionales de la infancia - permitirá a los profesionales de enfermería desarrollar una comunicación sencilla, empática, acogedora y humanizada en sus actuaciones asistenciales en las urgencias pediátricas. Por tanto, cuanto más sencilla y adecuada sea la comunicación entre los profesionales sanitarios y los cuidadores/familiares, más fácil les resultará comprender y acceder a la información sanitaria durante la recuperación y el tratamiento del niño^(1,24)^.

Las limitaciones de este estudio incluyen la evaluación inmediata y la asistencia de los participantes al final del proceso educativo, influenciadas por la escasez de personal, la naturaleza del servicio y la elevada carga de trabajo. Factores externos interferentes en entornos de hospitalización crítica y el escenario pandémico con el nuevo coronavirus pueden haber impactado en la implicación y participación de los profesionales de enfermería en las actividades realizadas durante el proceso educativo.

Involucrar a los profesionales en las actividades de formación continuada, que requieren más tiempo para la reflexión y la construcción del conocimiento, parece ser un reto para garantizar una mayor validez externa de los datos. Otra limitación fue el análisis de la comunicación sólo desde la perspectiva de los profesionales de enfermería, aunque reconocemos la importancia de las evaluaciones centradas en el niño y la familia para evaluar las repercusiones de las acciones de otros profesionales de la salud que componen este escenario.

## CONCLUSIÓN

Esta investigación ha aportado información relevante sobre los conocimientos y perspectivas de los profesionales de enfermería en un proceso educativo centrado en la comunicación y la AS en el contexto de una urgencia pediátrica. Como se ha observado, la comunicación de los profesionales de enfermería puede influir en la capacidad del niño/familia para comprender, evaluar y aplicar la información sanitaria para promover la AS.

Además de las estrategias lúdicas ya utilizadas en las urgencias pediátricas, como los dibujos animados, los accesorios y los uniformes con motivos infantiles, los participantes ampliaron sus habilidades de comunicación basadas en la AS, como el refuerzo del uso de juguetes terapéuticos, la técnica *teach-back* y las marionetas. Estas estrategias involucran a los niños en el diálogo y los motivan a participar en las actividades asistenciales.

Las actividades de enseñanza-aprendizaje estimularon la participación activa y reflexiva del equipo de enfermería, con un repertorio más amplio de conocimientos y habilidades de AS, especialmente en lo que se refiere a estrategias y enfoques de comunicación específicos para los niños. También reforzaron la relación de confianza entre los profesionales y el niño/familia. Los datos presentados pueden apoyar la aplicación de metodologías de enseñanza-aprendizaje en otros servicios de urgencias pediátricas, así como en otros entornos asistenciales en los que la comunicación en salud debe ser problematizada y ampliada a través de la reflexión sobre el trabajo.

## Data Availability

Los datos que respaldan los resultados de este estudio se publicaron en la disertación que dio lugar al artículo, disponible en: https://repositorio.ufpe.br/handle/123456789/46079.
